# Effect of pesticide exposure on liver function tests and serum cholinesterase levels among floriculture industry workers in Bahirdar, Ethiopia: a comparative cross-sectional study

**DOI:** 10.1038/s41598-026-51363-8

**Published:** 2026-05-03

**Authors:** Habtamu Molla Gietie, Gobena Dedefo, Mekdes Alem, Getachew Wolde, Mohammed Aliye, Abditsion Disani, Wessene Habtu, Genet Ashebir, Edmealem Minlarg, Tilahun Bitew, Abebe Edao, Mistire Wolde

**Affiliations:** 1 Department of Medical Laboratory Science, College of Medicine and Health Science, Debre Markos University, P.O. Box 269, Debre Markos, Ethiopia; 2 Department of Medical Laboratory Sciences, College of Health Sciences, Addis Ababa University, P.O. Box 1176, Addis Ababa, Ethiopia; 3 Department of Medical Laboratory Sciences, St. Paul Hospital Millennium Medical College, P.O. Box 1271, Addis Ababa, Ethiopia; 4 Department of Medical Laboratory Science, College of Medicine and Health Science, Werabe University, P.O. Box 1888, Werabe, Ethiopia; 5 Department of Medical Laboratory Sciences, Institute of Health, Bule Hora University, P.O. Box 144, Bule Hora, Ethiopia; 6 National References Laboratory for Clinical Chemistry, Ethiopian Public Health Institute, P.O. Box 1242, Addis Ababa, Ethiopia; 7 Department of Pharmacy, College of Medicine and Health Science, Debre Markos University, P.O. Box 269, Debre Markos, Ethiopia; 8 Department of Biomedical Science, School of Medicine, Debre Markos University, P.O. Box 269, Debre Markos, Ethiopia

**Keywords:** Floriculture, Horticulture, Liver function, Choline esterase, Diseases, Environmental sciences, Health care, Medical research, Risk factors

## Abstract

Workers in the floriculture industry are exposed to various classes of pesticides through skin contact, inhalation, and ingestion. Although many pesticides are known to exert hepatotoxic effects and inhibit serum butyrylcholinesterase (BChE), no prior studies have been conducted in Ethiopia, particularly in the Amhara region. This study aimed to assess the effects of pesticide exposure on liver function tests and BChE among floriculture workers in Bahir Dar, Ethiopia. A comparative cross-sectional study was conducted from February 8, 2025, to May 28, 2025, including 103 floriculture workers and 51 community controls from Zege town. Sociodemographic and clinical data were collected using structured questionnaires, and venous blood samples were analyzed using the Cobas 6000 analyzer. Data were entered in Epidata 4.7 and analyzed in SPSS version 27. Statistical analyses included chi-square/Fisher’s exact tests, t-tests, ANCOVA, one-way ANOVA, and multivariate logistic regression, with significance set at *p* < 0.05. Floriculture workers had significantly higher ALT (27.71 ± 0.44 U/L vs. 22.71 ± 0.47 U/L; MD = 4.99 [95% CI: 3.72, 6.25]; *p* < 0.001), AST (28.46 ± 0.68 U/L vs. 20.69 ± 0.73; MD = 7.77 [95% CI: 5.98, 9.74]; *P* < 0.001), TBL ( 0.831 ± 0.028 mg/dL vs. 0.362 ± 0.030 mg/dL; MD = 0.47 [95% CI: 0.39, 0.55]; *P* < 0.001), and DBL (0.298 ± 0.012 mg/dL vs. 0.170 ± 0.021 mg/dL; MD = 0.13 [95% CI: 0.09, 0.17]; *p* < 0.001) compared with controls. Conversely, total protein (7.385 ± 0.058 g/dL vs. 7.624 ± 0.098 g/dL; MD = − 0.24 [95% CI: −0.46, − 0.01]; *p* = 0.038), albumin (4.255 ± 0.052 g/dL vs. 4.623 ± 0.088 g/dL; MD = − 0.37 [95% CI: −0.57, − 0.17]; *p* < 0.001), A/G ratio (1.394 ± 0.040 vs. 1.580 ± 0.067; MD = − 0.19 [95% CI: −0.34, − 0.03]; *p* = 0.018), and BChE (5105 ± 141 U/L vs. 6699 ± 239 U/L; MD = − 1594 [95% CI: −2142.66, − 1045.39]; *p* < 0.001) were significantly lower among floriculture workers compared to control. Workers with more than 10 years of exposure showed markedly elevated ALT, AST, TBL, DBL, and globulin, while reduced albumin, A/G ratio, and BChE with < 5 years and 5–10 years (*p* < 0.05). Overall, prolonged pesticide exposure was associated with significant alterations of liver function and BChE among floriculture workers.

**Supplementary Information:**

The online version contains supplementary material available at 10.1038/s41598-026-51363-8.

## Introduction

Pesticides are chemical substances used to kill, control, or repel pests, and they are widely applied in agriculture to enhance crop protection and improve productivity^1^. Workers in floriculture often absorb pesticides by ingestion, inhalation, or skin contact, which are then transported through the circulatory system to impact different organs^2^. It is widely known that the liver plays a significant role in the detoxification of xenobiotics and related compounds through metabolism and excretion. Hence, xenobiotics like pesticides have the potential to cause significant damage to the liver^3^.

Globally, pesticide exposure is a major public health concern. According to data from the World Health Organization (WHO), there are between one and five million cases of poisoning among agricultural laborers annually, varying in severity and potentially affecting vital organs like the kidneys, lungs, or heart^4^. An estimated 11,000 people die globally each year as a result of unintentional pesticide poisoning. The maximum estimation of Unintentional Acute Pesticide Poisoning (UAPP)cases was observed in South and South-Eastern Asia, followed by East Africa, where Ethiopia is located^5^.

In Ethiopia, the floriculture industry began to emerge in the late 1990 s and has since become the second-largest flower exporter from Africa to the European Union (EU) after Kenya. The industry has created job opportunities for individuals with low educational levels and those living in poverty, contributing significantly to the country’s foreign currency earnings^6^. Pesticide use in agricultural sectors in Ethiopia has increased in recent years due to the rapid Growth of flower farms^7^. The import of pesticides in 2002–2006 was 13,381 tons, and this increased to 30,059 tons from 2006–2011^8^. Organophosphates(OPs) and carbamates (CMs) are commonly used pesticides on flower farms^9^.

Liver function tests (LFTs) are common screening blood tests. It includes: alanine aminotransferase (ALT), aspartate aminotransferase (AST), alkaline phosphatase (ALP), total protein, albumin, total bilirubin (TBL), direct bilirubin (DBL), and gamma-glutamyltransferase (GGT), and others. These enzymes and proteins change in the blood when liver cells are injured. ALT and AST mainly reflect hepatocellular damage, while ALP is most useful for detecting bile duct disorders. Elevated levels can signal inflammation or liver dysfunction. Pesticides, including insecticides, herbicides, and fungicides, are well known to be hepatotoxic^10–12^.

Blood cholinesterase testing is widely used to monitor exposure to CMs and OPs pesticides. True acetylcholinesterase (AChE) occurs in red blood cells and brain tissue, while butyrylcholinesterase (BChE) is produced in the liver and found in serum. Pesticide exposure is strongly linked to reduced cholinesterase levels^13^. The BChE might be a more sensitive indicator of exposure to some OP compounds such as malathion, diazinon, and dichlorvos^14^. The effect of OP exposure on cholinesterase activity was found predominantly in BChE. One of the possible reasons is that the potential inhibition of AChE and BChE varies widely among the different OP compounds. Moreover, some OPs inhibit BChE more strongly than AChE. The inhibition of BChE is highly correlated with intensity and duration of higher exposure to a large group of OP and CB pesticides^15^.

The use of pesticides is extremely harmful to plants, animals, humans, and the abiotic component^16^. Many studies using lab animals have demonstrated the hepatotoxic effects of agricultural pesticides^17,18^. There have been reports of farmers from Palestine, Thailand, Iraq, Brazil, and India experiencing altered liver function^19–23^. Additional studies also found a correlation between liver dysfunction indicators and pesticide exposure^24,25^.

Pesticide hepatotoxicity results from the generation of reactive oxygen species (ROS) and reactive intermediates upon exposure^26^. The production of free radicals and oxidative stress can be significant factors in pesticide toxicity^27,28^. The mitochondria are primarily responsible for the induction of oxidative stress. Electron leakage from the respiratory chain leads metabolism and ATP production, causes apoptotic pathways, and produces ROS^29,30^. Numerous liver disorders, such as chronic hepatitis, steatosis, ischemia injuries, aging, and inflammatory damage, are mostly caused by mitochondrial oxidative damage^31,32^.

Though several studies across the world pointed to the risk of occupational exposure to pesticides on liver and renal function profiles, to the best of the principal investigator’s knowledge, there is no published data that is similar to this study in Ethiopia in general, in the study area in particular. Therefore, this study aimed to assess the effect of pesticide exposure on the liver using LFTs (ALT, AST, ALP, total protein, Albumin, Globulin, DBL, TBL, and A/G ratio) and BChE in floriculture industry workers. To address this gap, this research was done to evaluate the LFTs and BChE levels of workers and identify potential risk factors associated with their occupational environment.

## Methods

### Study area, design, and period

A comparative cross-sectional study was conducted at Tana Flora Floriculture Industry, Bahir Dar, Ethiopia, from February 8, 2025, to April 28, 2025. Data for the control group were collected in Zege town from April 29, 2025, to May 28, 2025. Bahirdar is located 563 km from Addis Ababa, Ethiopia’s capital. Both rural and urban residents reside in this city. Four private hospitals, 56 private specialty clinics, 13 private medium clinics, 11 healthcare facilities, 15 health posts, and three public hospitals can all be found in the city^33^. Geographically, Tana Flora is located at a latitude of 11°35’30” North and a longitude of 37°23’30” East. It is located in the Amhara Region of Ethiopia’s Wonjeta Kebele, Bahir Dar Zuria region. On June 22, 2009, Tis Isat Water Works and Gafat Endowment founded it. The distances are 582 km to Addis Ababa and 18 km to Bahir Dar. The area has 124 hectares, 40 of which are allocated for growing fresh cut roses in greenhouses for the world’s flower industry. Because of floriculture, 950 people now have jobs, 70% of them are women. It contributes to the country’s economic growth by selling cut flowers, bringing in an average of $10 million USD annually^34,35^.

The controls were selected from the small town of Zege. Zege is a rural satellite town of the Bahr-Dar city government, located 32 km from the Amara National Regional capital, Bahir Dar^36^. According to the city government of Bahir Dar’s 2019 population projection, Zege now has 10,083 residents (4041 men and 6042 women). Zege has three health posts and one health center, and each of these facilities provides medical care to the local population as well as those in the surrounding area. The Tana Flora floriculture company is 15 km away from Zege rural town^37^. The study area map is found in Supplementary information (Figure S1).

### Population

#### Source population

- Exposed group- All employees of Tana Flora Floriculture Industry.
- Control- All apparently healthy residents of Zege town.

#### Study population

- Exposed- All active employees of Tana Flora Floriculture Industry.
- Control- All volunteer age and sex matched residents of Zege town.

### Eligibility criteria

#### Inclusion criteria

Exposed group: Workers who have been working a minimum of six months in the floriculture site and are permanently employed. Workers who voluntarily participated in the study and signed the consent form.

Control group: Apparently healthy residents of the Zege satellite town who are volunteers and matched with exposed participants in age, sex, and not exposed to pesticides.

#### Exclusion criteria

Exposed group: Workers who have a history of liver health problems, chronic liver disease pregnant women, Medications affect LFTs, Administrative staff, and managers.

Control group: Residents with a history of liver health problems, chronic liver disease, Medications affect LFTs, pregnant women,

### Variables

#### Dependent variable

- Liver function tests.
- Serum cholinesterase.

#### Independent variables

Socio-demographic characteristics (age, sex, education, marital status), duration of work exposure, personal protective measures, types of pesticides, alcohol consumption, coffee consumption, **and** Body Mass Index (BMI).

### Sample size determination

A two independent means formula was used to determine the sample size of exposed group and controls under the following assumptions: a 95% confidence level, Power (1-β) − 80% (0.80), ratio of exposed to control 2:1. The mean and standard deviation (µ ± SD) of ALT in exposed and control is (27.38U/L ± 9.08 U/L) and (23.71 ± 7.58), respectively from Indian study^23^. The sample size was 118 for the pesticide-exposed and 59 for the control. The total active floriculture workers of the company were 490, which is less than 10,000. The sample size was calculated using the Sample reduction formula. The sample size was 95 pesticide-exposed participants and 47 controls, respectively. To compensate for the non-respondents and to minimize errors probably arising from the likelihood of non-compliance, 10% was added, giving a final sample size of 105 for pesticide-exposed participants and 52 for the control.

### Sampling techniques

The exposed groups were recruited for the investigation using stratified sampling and proportional simple random sampling. There were 376 women and 215 men working in the floriculture sector. 37 employees (25 men and 12 women) were excluded from the study because they were supervisors and administrative staff. 64 workers (24 men and 40 women) were excluded from the study because they were temporary employees with fewer than six months of employment. The study included 490 permanent labor workers. Of the 490 workers, 320 came from the greenhouse (232 females and 88 men), 126 from packing (89 females and 37 males), 33 from spraying (all males), and 11 from irrigation (all males). 105 pesticide-exposed participants were recruited for the study. Two Greenhouse participants were excluded from the study due to known liver disease. In proportion, there were 27 (8 males and 19 females) from the packing house, 65 (47 females and 18 males) from the greenhouse, 8 from spray (all males), and 3 from irrigation (all males). There were 66 females and 37 males in the final sample of 103 (Figure S2). Controls were selected using a purposive sampling technique from households in Zege town across three kebeles (the smallest administrative units of the government). A total of 18 households were selected from Kebele 1, 17 from Kebele 2, and 17 from Kebele 3 using simple random sampling. From each selected household, one participant was purposively recruited and matched with a case by age and sex. Initially, 52 controls (34 females and 18 males) were enrolled in the study. However, one female participant with a known history of liver disease was excluded. Consequently, the final control group consisted of 51 participants (33 females and 18 males).

### Data collection methods and procedures

#### Sociodemographic and associated factors

Socio-demographic data and associated factors were collected from study participants by face-to-face interview using a questionnaire. The questionnaires were adopted and modified from different literature in the English language and were translated into local languages by interviewers to facilitate the understanding of interviewees and to limit the bias of data collection. Then, using a translated questionnaire, the study data were collected by face-to-face interviews and self-administered questionnaires. The questionnaire consisted mainly of closed and open-ended questions. It was focused on socio-demographic data, duration of employment, protective methods, health-related problems, pesticide-related symptoms, and coffee and alcohol drinking status. The weight of individuals was measured with digital balances, and the height of individuals was measured in M. BMI was calculated from weight in Kg and height in m².

#### Specimen collection and processing

Following written informed consent and response to the questionnaire, the participants were registered in the notebook, and a serial number was given to each. Blood samples from study participants were collected. Then, blood samples were collected from each participant based on his or her voluntarism. 5 ml of venous blood was collected in an SST test tube from the medial cubital of the forearm with swabbing by gauze or cotton moistened with 70% alcohol by the principal investigator and trained laboratory professional. The whole blood sample was stored at room temperature for 10–20 min until it coagulated. Then the blood sample was transported to the laboratory department of International Clinical Laboratories (ICL) with an ice-cold box within 4 h and centrifuged at 3,000 revolutions per minute for 5 min to separate the serum from the red cells. After centrifugation, the serum was separated from the gel and was placed in a Nunc tube in a deep freezer at −70 °C in the laboratories. After all specimens were collected and preserved in a deep freeze, all the samples were transported and analyzed at the Ethiopian Public Health Institute (EPHI). The specimen will be analyzed by the principal investigator and EPHI National Reference Clinical Chemistry laboratory workers. Liver function (ALT, AST, ALP, TBL, Total protein, Albumin, DBL, and BChE were done on the EPHI National Clinical Chemistry Reference Laboratory by Cobas 6000 clinical chemistry analyzer.

### Laboratory analysis

#### Liver Function tests (LFTs)

Liver parameters, including ALT, AST, and ALP, Total protein, TBL, DBL, and Albumin, were analyzed in the serum. These biochemical parameters were determined by spectrophotometric determination of their absorbance using analytical grade laboratory reagent kits. The laboratory reagent kits from Roche were used to assess the concentration of ALT (IFCC without pyridoxal phosphate method)^38^, AST (IFCC without pyridoxal phosphate method)^38^, ALP(IFCC P-Nitro Phenyl Phosphate substrate method)^39^, Total protein (Biuret reaction method)^40^, TBL (Diazo reaction)^41^, DBL (Diazo reaction)^42^, and Albumin (BCG method)^43^ in the serum. All LFTs were analyzed by the Cobas 6000 automated clinical chemistry analyzer with its own reagent kit. All biochemical analyses for this study were performed according to the manufacturer’s protocol. The details of laboratory test principles of LFTs and Laboratory procedure is provided in (Supplementary file 2).

#### Serum cholinesterase (BChE)

BChE was measured by the Schmidt et al. method^44^ in a Cobas 6000 automated clinical analyzer by spectrophotometric determination of its absorbance using analytical grade laboratory reagent kits. The laboratory reagent kit from Roche was used to assess the activity of serum cholinesterase. It was analyzed by its own reagent kit. The details of the laboratory test principle of BChE is provided in (Supplementary file 2).

#### Interpretation of results

The results were interpreted by using the normal reference range of test kits as follows. Therefore, after the test had been analyzed, the result for each analyte was interpreted based on the Adult reference range. The reference ranges of each laboratory test is provided in (Supplementary file 2).

### Data quality assurance

The quality of the data was assured by using a validated and pretested questionnaire. Before the actual data collection, pre-testing was done on 5% of the total study subjects at the Ethio Agriceft Floriculture industry, which was not included in the actual study, and based on the findings, necessary amendments were made. Data collectors were trained for one day intensively on the study instrument and data collection procedure, which included the relevance of the study, the objective of the study, confidentiality of the information, informed consent, and interview technique. The data collectors were working under the close supervision of the supervisors to ensure adherence to correct data collection procedures. The principal investigator reviewed the filled questionnaires at the end of data collection every day for completeness. The principal investigator and the data collectors were to conduct a morning session to solve the problem if encountered, as early as possible, and to take corrective measures accordingly. Moreover, the data were carefully entered and cleaned before the analysis.

The test was analyzed EPHI National Clinical Reference Laboratory. The equipment was calibrated monthly by the type-Auto calibrator. Besides, two levels (normal and pathological) of internal quality control (IQC) samples were run along with the serum sample. The EPHI National Clinical Chemistry Reference Laboratory received accreditation from ENAO on February 17, 2022. The accreditation is awarded to 16 tests, which are ALT, AST, ALP, LDH, Albumin, Alpha amylase, Cholesterol, TG, Urea, Creatinine, Total protein, Uric acid, Glucose, TBL, DBL, and Phosphate in accordance with the requirements of ISO 15,189: 2013. The control sample results were interpreted using the Westgard multi-rule algorithm. The sample was analyzed after thoroughly understanding the leaflet for each analyte by the principal investigator and senior laboratory technologists.

After the test was analyzed by using the selected method, the printed result was checked for all post-analytical factors, like the unit of reporting and the serial number given by the investigator. And the result was approved by the responsible laboratory technologist in the laboratory. Then the printed result was immediately given to the principal investigators.

#### Operational definitions

##### BMI:

was considered from weight and height measurement and categorized as < 18.5 kg/M^2^ (Underweight), 18.5–24.9 kg/M^2^ (Normal), 25.0–29.9 kg/M^2^ (Overweight), and ≥ 30 kg/m^2^ (Obese)^45^.

##### LFT abnormality:

It was defined as any parameters of the liver enzymes, TBL, DBL, and globulin, Total protein, albumin and A/G ratio greater than upper limit of normal reference range, while, decrease Total protein, albumin, and A/G ratio lower than the lower limit of the normal reference range(i.e. ALT greater than or equal to 41.00 U/L and 33.00 U/L for male and female, respectively; AST greater than or equal to 40.00 U/L and 32.00 U/L for male and female, respectively; ALP greater than 129 U/L and 104 U/L for male and female respectively; TBL greater than or equal to 1.4 mg/dl and 0.9 mg/dl male and female, respectively; DBL greater than or equal to 0.3 mg/dl for adult male and female; Total protein less than 6.6 mg/dl or greater than 8.7 mg/dl for male and female; Albumin less than 3.5 mg/dl or greater than 5.2 mg/dl for male and female; Globulin greater than 3.50 mg/dl or less than 2 mg/dl for male and female; Albumin to globulin ratio less than 1.0 or greater than 2 for adult male and female^46^.

##### BChE level:

Low BChE level – BChE less than 5320 U/L and 4260 U/L for adult males and females, respectively; Normal BChE level – BChE between 5320 U/L and 12920U/L and 4260 U/L to 11,250 U/L for adult males and females, respectively; High BChE level – BChE greater than 12,920 U/L and 11250U/L for adult males and females, respectively^47^.

##### Apparently Healthy:

An individual who has no signs, symptoms, or history of any disease at the time of assessment.

### Data analysis and interpretation

Data was cleared, edited, checked for completeness manually, and will be entered into Epidata 4.7, and analyzed using the Software Package for the Social Science (SPSS) for version 27 for Windows^®^ (SPSS Inc., Chicago, IL, the USA) for analysis. After organizing and cleaning the data, frequencies and percentages will be calculated for all variables that are related to the objectives of the study. Categorical variables were analyzed using the chi-square. An independent t-test will be used to determine the mean difference between pesticides exposed male and females. General linear mode (GLM) with univariate analysis of covariance (ANCOVA) to adjust for cofounding socio-demographic variables and compare pesticide-exposed and control groups. One-way ANOVA (Analysis of variance) was used to determine the duration exposure category of the exposed group with liver function tests, and serum Cholinesterase. The Kruskal-Wallis test was used to determine the median difference between pesticides exposed groups. Bonferroni or tukey correction was applied to adjust for multiple pairwise comparisons where appropriate. To assess the normality of the data, both the Kolmogorov-Smirnov and Shapiro-Wilk tests were employed, with *p* > 0.05, indicating a normal distribution. A multicollinearity test was carried out to see the correlation between independent variables using the variance inflation factor (VIF), and no variables were observed with a VIF of > 2, indicating the non-existence of multicollinearity among the variables in this study. The model’s goodness of fit was evaluated using the Hosmer and Lemeshow test (*p* > 0.05), which was found to be insignificant. Homogeneity of regression slopes was evaluated by testing the interaction between the covariate and the group variable. The interaction term was statistically not significant in most of dependent variables (*p* > 0.05), indicating that the assumption was satisfied. In some dependent variables interaction term was statistically significant (*p* < 0.05), indicating that the homogeneity of regression slopes assumption was violated. Therefore, the interaction term was retained in the model to account for differential relationships between the covariate and the outcome variable across groups. Binary logistic regression was used to identify significant variables. Those with a p-value < 0.25 in the bi variable analysis were included in multivariable regression to control for confounders. A P-value of less than 0.05 was considered statistically significant. In a multivariable logistic regression model, an adjusted odds ratio (AOR) with a 95% confidence interval (CI) was reported to measure the strength of association. Finally, the results were presented using tables and other narrative forms.

## Results

### Socio-demographic characteristics

A total of 154 study participants were included from 157 participants in the study, with a response rate of 98%, including 103 pesticide-exposed participants and 51 controls. The majority of the pesticide-exposed group was females, 66/103 (64.1%). There was no significant difference in sex, age categories, and marital status between case and control (*p* > 0.05). However, significant differences were observed in educational status, monthly income, and residency (*p* < 0.001). A greater proportion of controls had attained secondary education or higher, resided in urban areas, and earned more than 5000 ETB monthly (Table 1).

**Table 1:** Sociodemographic characteristics of study participants.

Sociodemographic characters	Category	Group		Total (*N* = 154) No. (%)	χ² (P-Value)
		Case (*N* = 103) No. (%)	Control (*N* = 51 No. (%)		
Sex	Male	37(35.9)	18(35.3)	55(35.7)	0.006(0.939)
	Female	66(64.1)	33(64.7)	99(64.3)	
Age (Years)	18–25	63(61.2)	34(66.7)	97(63)	0.443(0.506)
	26–32	40(38.8)	17(33.3)	57(37)	
Marital status	Married	52(50.5)	25(49)	77(50)	5.275(0.153)
	Single	42(40.8)	26(51)	68(44.2)	
	Windowed	1(1)	0(0)	1(0.6)	
	Divorced	8(7.8)	0(0)	8(5.2)	
Level of education	Illiterate	39(37.9)	3(5.9)	42(27.3)	**23.741(< 0.001)**
	Reading and Writing	20(19.4)	8(15.7)	28(18.2)	
	Elementary	19(18.4)	11(21.6)	30(19.5)	
	Secondary school	16(15.5)	17(33.3)	33(21.4)	
	Diploma/university above	9(8.7)	12(23.5)	21(13.6)	
Monthly income(ETB)	1500–3000	50(48.5)	4(7.8)	54(35.1)	**42.915(< 0.001)**
	3000–5000	44(42.7)	21(41.2)	65(42.2)	
	> 5000	9(8.7)	26(51)	35(22.7)	
Residency	Urban	21(20.4)	36(70.6)	57(37)	**36.871(< 0.001)**
	Rural	82(79.6)	15(29.4)	97(63)	
BMI	< 18.5 (Underweight)	8(7.8)	9(17.6)	17(11)	5.319(0.077)
	18.5–24.9 (Normal)	91(88.3)	42(82.4)	133(86.4)	
	25–30 (Overweight)	4(3.9)	0(0)	4(2.6)	

Notes: χ²- Chi square, BMI-Body Mass Index, ETB-Ethiopian Birr.

### Occupational history, working environment, and health perceptions among pesticide-exposed floriculture workers

**Fig. 1:**
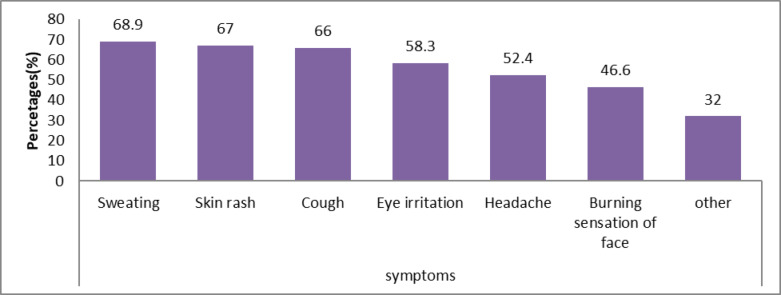
Pesticide-induced symptoms among floriculture workers during the past 6 months (*n* = 103).

In this study, 35/103 (34%) had worked in horticulture for less than 5 years, while 48/103 (46.6%) had 5–10 years of experience, and 20/103 (19.4%) had worked for more than 10 years. Common symptoms reported during work in the past 6 months included sweating 71/103 (68.9%), skin rash 69/103 (67.0%), cough 68/103 (66.0%), eye irritation 60/103 (58.3%), headache 54/103 (52.4%), others (Fatigue, back pain and diarrhea) 33/103(32%) (Fig. 1). About 8/103(7.8%) study participants reported experiencing foot edema, all of which occurred after employment. About 65/103 (63.1%) of the floriculture workers worked in greenhouses, 27/103 (26.2%) in packing areas, 8/103 (7.8%) in spraying, and 3/103 (2.9%) in irrigation (Table 2).

**Table 2:** Occupational history, working environment, and health perceptions among pesticide-exposed floriculture workers.

Variables	Categories	Frequencies	Percentages
Work experience	< 5years	35	34
	5–10 years	48	46.6
	> 10 years	20	19.4
Mean work experience (years)	Mean ± SD	6.85 ± 3.19	/
Do you have foot edema?	Yes	8	7.8
	No	95	92.2
If your answer is yes, was the foot edema present before you were employed in this company?	Yes	0	0
	No(Occurred after employment)	8	100
Current health perception	Excellent	6	5.8
	Very good	20	19.4
	Fear	36	35
	Poor	26	25.2
	Very poor	15	14.6
Working environment	Green house	65	63.1
	Packing	27	26.2
	Spraying	8	7.8
	Irrigation	3	2.9

### Types of pesticides used in the study area

About 34 pesticides were used during survey in the floriculture industry, 18 (53%) of pesticides were grouped under unlikely to cause hazard, WHO Hazard Class (Class U), but 16(43%) grouped into hazardous classes, from those 13(38%) grouped under class II, 2(6%) class Ib and 1(3%) class III. Most of the active compounds belong to organophosphates, spinosyns, neonicotinoids, and morpholines, with widespread use of insecticides and fungicides in the horticultural sector (Table 3).

**Table 3:** Types of pesticides used in the study area.

No	Brand Name	Active Ingredient(s)	Pesticide Group	Chemical Group/Class	WHO Hazard Class
1	Amino mite	Organosilicon 100%	Miticide	Organosilicon	U
2	Mite sweep 100EC	Hexythiazox 100 g/l	Miticide	Oxazoline	U
3	Efentizine	Clofentazine	Acaricide	Carbamate	II
4	ACE 75SP	Acephate 750 g/kg	Insecticide	Organophosphate	II
5	Orthene 97% Pellet	Acephate 970 g/kg	Insecticide	Organophosphate	II
6	Delegate 250 WG	Spinetoram 250 g/kg	Insecticide	Spinosyn	U
7	Radiant 120 SC	Spinetoram 120 g/l	Insecticide	Spinosyn	U
8	Teppeki	Flonicamid 150 g/kg	Insecticide	Pyridinecarboxamide	U
9	Tracer 485 SC	Spinosad 485 g/l	Insecticide	Spinosyn	U
10	Hinder 500 SP	Thiocyclam hydrogenoxalate 500 g/kg	Insecticide	Oxime carbamates	II
11	Belloxam 30%	Thiamethoxam 300 g/l	Insecticide	Neonicotinoid	II
12	Actara 250 WG	Thiamethoxam 250 g/kg	Insecticide	Neonicotinoid	II
13	Acepride 200 WSP	Acetamiprid 200 g/l	Insecticide	Neonicotinoid	II
14	Agent 50 SC	Fipronil 500 g/l	Insecticide	Phenylpyrazole	II
15	Echo 1.92 EC	Emamectin benzoate 192 g/l	Insecticide	Avermectin	U
16	Meltatox 385 EC	Dodemorph acetate 385 g/l	Fungicide	Morpholine	U
17	Collis 300 SC	Boscazid 200 g/l + Kresoxim methyl 100 g/l	Fungicide	Benzimidazole +SDHI	U
18	Climate 500 SC	Kresoxim methyl 500 g/l	Fungicide	SDHI	U
19	Limpulse 500 EC	Spiroxamine 500 g/l	Fungicide	Morpholine	U
20	Splendor 500 SC	Spiroxamine 500 g/l	Fungicide	Morpholine	U
21	Sprinox 500 EC	Spiroxamine 500 g/l	Fungicide	Morpholine	U
22	Sulphergold 80% WDG	Sulphur 800 g/kg	Fungicide/Miticide	Elemental Sulfur	U
23	Runner 240 SC	Methoxyfenozide 240 g/l	Insecticide	Diacylhydrazine	U
24	Match	Infenuron	Insecticide	Chloronicotinyl	II
25	Diazinox 60 EC	Diazinon 600 g/l	Insecticide	Organophosphate	Ib
26	Diazol 60 EC	Diazinon 600 g/l	Insecticide	Organophosphate	Ib
27	Agrilax	Dimethoate 40% EC	Insecticide	Organophosphate	II
28	Aliette flash	Fosetyl aluminum 800 g/kg	Fungicide	Phosphonate	U
29	Vital	Potassium phosphate	Fungicide	Phosphate compound	U
30	Previcur ESL	Propamocarb hydrochloride 66.5%	Fungicide	Carbamate	U
31	Agridif	Difenoconazole 250 g/l	Fungicide	Triazole	II
32	Jafromax	Difenoconazole 250 g/l	Fungicide	Triazole	II
33	Glyphosate	Glyphosate	Herbicide	Organophosphonate	III
34	Malathion	Malathion	Insecticide	Organophosphate	II

Note: Ia = Extremely hazardous, Ib = Highly hazardous, II = moderately hazardous, III = slightly hazardous, U = Unlikely to present acute hazard.

### Safety Practices of study participants among floriculture workers

Of the study participants, 38/103 (36.9%) reported PPE during work. For those not using PPE, the main reason was lack of supply of PPE, 49/103 (47.6%), followed by carelessness, 9/103 (8.7%), discomfort 5/103(4.9%), and the perception that PPE was unnecessary 2 (1.9%). Only 16 (15.5%) took a bath after pesticide application, and while 80/103 (77.7%) reported hand washing after work, only 42 (40.8%) changed clothes before going home. About 31/103(30.1%) of study participants ate/drank in the working area. Additionally, 44/103(42.7%) of study participants consumed coffee, and 35/103(34%) consumed alcohol. About 71/103 (68.9%) would visit a health facility, 22/103 (21.4%) stopped work, and the rest relied on traditional medicine, 10/103 (9.7%). Only 24/103(23.3%) of study participants took a periodical checkup (Table 4).

**Table 4:** Safety Practice of study participants among floriculture workers.

Variables	Category	Frequency	Percentiles (%)
Do you use PPE?	Yes	38	36.9
	No	65	63.1
If yes, which PPE have you used at your work? ^a^	Glove	38	36.9
	Hat	18	17.5
	Mask	29	27.6
	Special shoes	31	30.1
	Gown	33	32
	Eye goggle	11	10.7
If you have not worn any of the equipment listed above, what is the reason?	Not provided	49	47.6
	Carelessness	9	8.7
	Not comfortable	5	4.9
	Not necessary	2	1.9
Do you take a bath after pesticides are applied?	Yes	16	15.5
	No	87	84.5
Do you wash your hands immediately after work?	Yes	80	77.7
	No	23	22.3
Do you change clothes before going home?	Yes	42	40.8
	No	61	59.2
Do you have the habit of eating/drinking in the working Area?	Yes	31	30.1
	No	72	69.9
Do you consume coffee?	Yes	44	42.7
	No	59	57.3
Do you consume alcohol?	Yes	35	34
	No	68	66
What action should be taken after pesticide exposure?	Traditional Medicine	10	9.7
	Stop work and rest	22	21.4
	Go to the health facility	71	68.9
Do you take a periodic checkup?	Yes	24	23.3
	No	79	76.7

Note: a- Multiple response is allowed.

### Use of personal protective equipment among pesticide-exposed groups

**Fig. 2:**
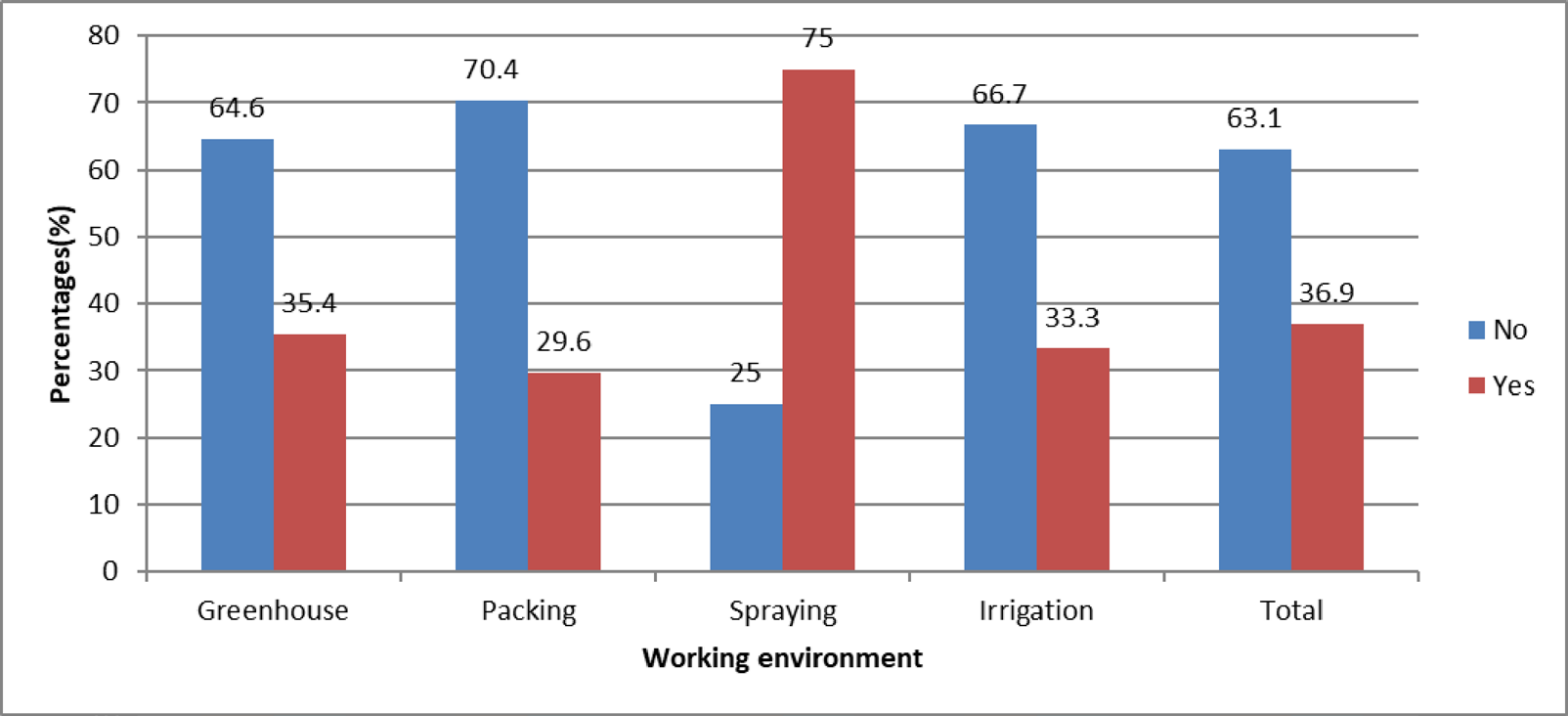
Use of personal protective equipment among pesticide-exposed groups.

**Fig. 3:**
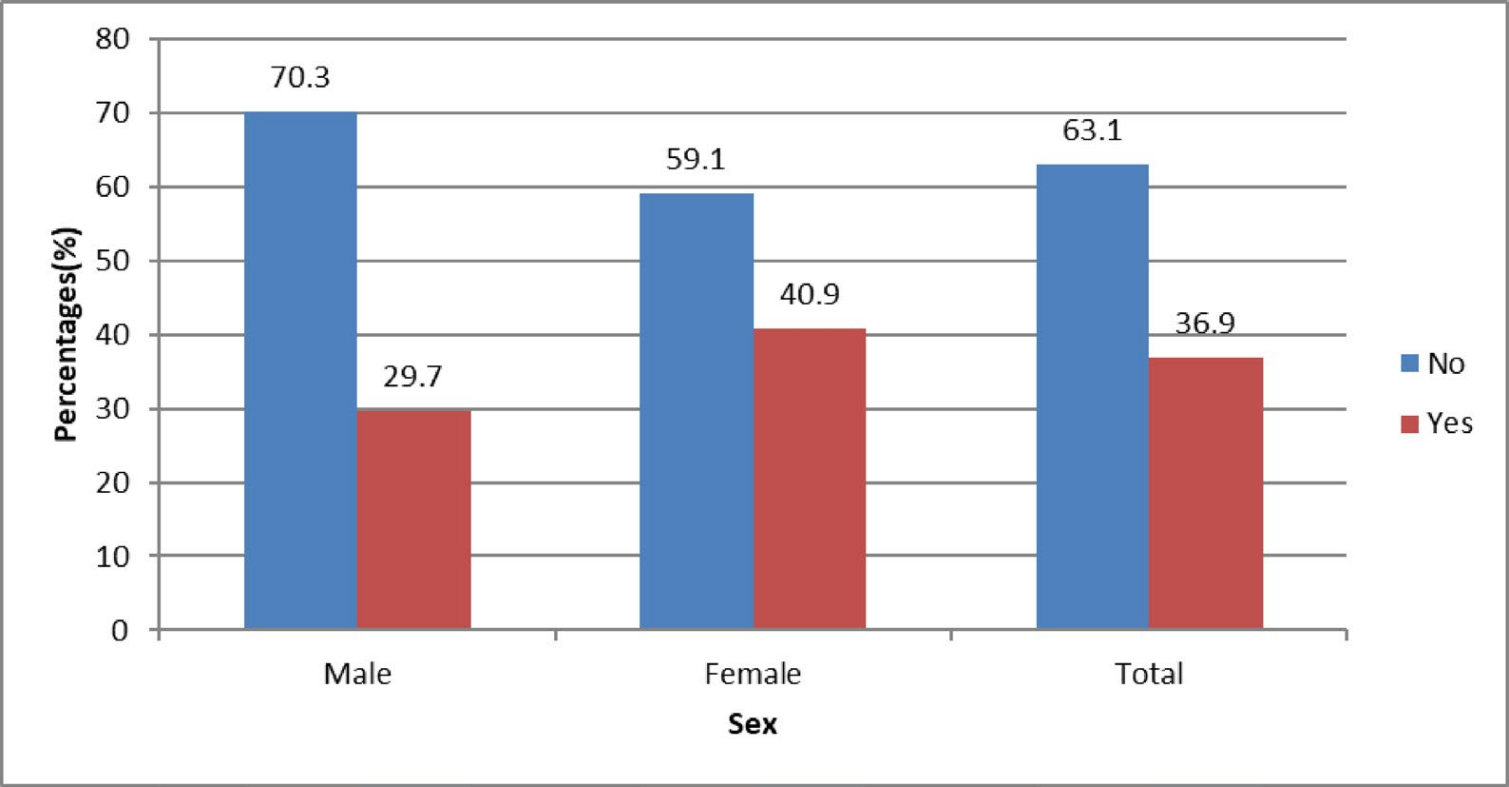
Use of personal protective equipment among male and female pesticide-exposed study participants.

PPE utilization showed significant variation by work environment. The highest level of PPE use was observed among spraying workers (75.0%), whereas PPE use was consistently low in greenhouse (35.4%), packing (29.6%), and irrigation (33.3%) activities. Overall, 36.9% reported PPE use, indicating widespread non-compliance (63.1%) across most working environments (Fig. 2). When stratified by sex, 29.7% of males reported PPE use compared with 40.9% of females, indicating higher compliance among female workers (Fig. 3).

### Comparison of liver function tests and serum cholinesterase level among pesticide-exposed and controls

ANCOVA was conducted to examine differences in LFTs and BChE levels between pesticide-exposed groups and controls, adjusting for sex, age, income, and residency. Pesticide exposure was significantly associated with elevated ALT, AST, TBL, and DBL, while reduced albumin A/G ratio and BChE, while ALP and globulin remained unaffected. Covariates such as age, sex, and income influenced some outcomes. The details of ANCOVA are provided in supplementary file 3 (Table S2).

The adjusted mean of ALT level was significantly higher in the case group (27.71 ± 0.44U/L) compared to the control group (22.71 ± 0.47 U/L), with *p* < 0.001. Similarly, the mean of AST was markedly elevated among cases (28.46 ± 0.68 U/L) compared to controls (20.69 ± 0.73U/L), with *p* < 0.001. TBL was 0.831 ± 0.028 mg/dL among cases compared to 0.362 ± 0.030 mg/dL in controls, with *p* < 0.001. DBL showed a similar trend (0.298 ± 0.012 vs. 0.170 ± 0.021 mg/dL; *p* < 0.001). A significant reduction was observed in serum albumin among exposed workers (4.255 ± 0.052 g/dL) compared to controls (4.623 ± 0.088 g/dL), with *p* < 0.001. Total protein was also significantly reduced in pesticide-exposed (7.385 ± 0.058) compared to controls (7.624 ± 0.098) with *p* = 0.038. The albumin-to-globulin (A/G) ratio was significantly lower in the case group (1.394 ± 0.040) compared to controls (1.580 ± 0.067), with *p* = 0.018. BChE levels were significantly decreased in cases (5105 ± 141 U/L) versus controls (6699 ± 239 U/L), with *p* < 0.001 (Table 5).

**Table 5:** Comparison of liver function tests and BChE level among pesticide-exposed and controls adjusted for sex, age, income and residency.

Parameters	Case	Control	Mean Difference	95% CI of mean difference (MD)	F value	*P* value	Partial eta squared(η²*P*)
	Adjusted Mean ± SE	Adjusted Mean ± SE					
ALT(U/L)	27.71 ± 0.44	22.71 ± 0.47	**+ 4.99**	**[3.72**,** 6.25]**	**59.61**	**< 0.001**	**0.328**
AST(U/L)	28.46 ± 0.68	20.69 ± 0.73	**+ 7.77**	**[5.98**,** 9.74]**	**60.957**	**< 0.001**	**0.333**
ALP (U/L)	80.04 ± 0.94	78.76 ± 1.00	+ 1.28	[−1.43, 3.99]	0.88	0.351	0.007
TBL(mg/dL)	0.831 ± 0.028	0.362 ± 0.030	**+ 0.47**	**[0.39**,** 0.55]**	**130.92**	**< 0.001**	**0.518**
DBL(mg/dL)	0.298 ± 0.012	0.170 ± 0.021	**+ 0.13**	**[0.09**,** 0.17]**	**27.968**	**< 0.001**	**0.243**
Total protein(g/dL)	7.385 ± 0.058	7.624 ± 0.098	**−0.24**	**[−0.47**,** −0.01]**	**4.367**	**0.038**	**0.030**
Albumin(g/dL)	4.255 ± 0.052	4.623 ± 0.088	**−0.37**	**[−0.57**,** − 0.17**	**13.101**	**< 0.001**	**0.084**
Globulin (g/dL)	3.130 ± 0.057	3.000 ± 0.097	+ 0.13	[−0.09, 0.35]	1.333	0.250	0.009
A/G ratio	1.394 ± 0.040	1.580 ± 0.067	**−0.19**	**[−0.34**,** −0.03]**	**5.713**	**0.018**	**0.039**
BChE(U/L)	5105 ± 141	6699 ± 239	**−1594**	**[−2142.66**,** − 1045.39]**	**32.988**	**< 0.001**	**0.189**

Notes: SE- Standard error, Generalized linear model is used for comparison, and P-value ≤ 0.05 is considered significant, Mean is adjusted for age, sex, income and residency.

### Comparison of liver function tests and serum cholinesterase among pesticide-exposed males and females

Comparison of LFTs and BChE between pesticide-exposed males and females showed that most parameters were similar between sexes. ALT, ALP, TBL, DBL, total protein, albumin, globulin, and A/G ratio did not differ significantly (all *p* > 0.05). However, AST was significantly higher in males (31.17 ± 5.31) compared to females (26.90 ± 5.08 U/L) with *p* < 0.001. BChE also significantly higher in males (5573.7 ± 931.2) compared with females (5092.9 ± 1014.6 U/L) with *p* = 0.019(Table 6).

**Table 6:** Comparison of liver function tests and BChE level among pesticide-exposed male and females.

Parameter	Male (*n* = 37)	Female (*n* = 66)	Mean Difference	95%CI of mean difference(MD)	t	*p*-value	Cohen’s d
	Mean ± SD	Mean ± SD					
ALT (U/L)	28.05 ± 3.51	27.41 ± 3.24	0.64	[−0.72, 1.99]	0.93	0.355	0.191
AST(U/L)	31.17 ± 5.31	26.90 ± 5.08	4.27	[2.17, 6.37]	4.03	**< 0.001**	**0.827**
ALP (U/L)	79.03 ± 6.06	79.13 ± 6.40	− 0.10	[−2.66, 2.46]	−0.08	0.937	−0.016
TBL(mg/dL)	0.87 ± 0.21	0.88 ± 0.22	−0.07	[−0.09, 0.08]	−0.17	0.869	−0.034
DBL(mg/dL)	0.29 ± 0.12	0.27 ± 0.10	0.02	[− 0.027, 0.06]	0.76	0.451	0.155
Total protein (g/dL)	7.46 ± 0.47	7.49 ± 0.45	−0.03	[−0.22, 0.16]	−0.32	0.748	−0.066
Albumin (g/dL)	4.44 ± 0.43	4.32 ± 0.45	0.12	[−0.06, 0.30]	1.29	0.201	0.264
Globulin (g/dL)	3.03 ± 0.41	3.17 ± 0.44	− 0.15	[−0.32, 0.027]	−1.67	0.098	−0.343
A/G ratio	1.50 ± 0.30	1.40 ± 0.31	0.10	[−0.023, 0.228]	1.62	0.109	0.332
BChE(U/L)	5573.7 ± 931.2	5092.9 ± 1014.6	480.8	[79.22, 882. 38]	2.38	**0.019**	**0.488**

Notes: SD- Standard deviation, independent t-test is used for comparison, CI- confidence interval, and P-value ≤ 0.05 is considered significant.

### Comparison of liver function tests and serum cholinesterase between pesticide-exposed groups

A Kruskal-Wallis test was used to compare the median of LFTs and serum cholinesterase across the working environment. A statistically significant difference was observed only for TBL levels (H = 16.59, *p* = 0.001). Post-hoc pairwise comparisons with Bonferroni adjustment showed that packing workers had significantly higher total bilirubin levels compared with greenhouse workers and spraying workers, with adjusted *p* < 0.05. In contrast, no statistically significant differences were found across working environments for ALT, AST, ALP, DBL, TBL, albumin, globulin, A/G ratio, and BChE (all *p* > 0.05(Table 7).

**Table 7:** Comparison of liver function tests and serum cholinesterase between pesticides exposed groups.

Parameters	Pesticide-exposed groups					
	Greenhouse	Packing	Spraying	Irrigation	H (df = 3)	*p*-value
	Median ± IQR	Median IQR	Median ± IQR	Median ± IQR		
ALT(U/L)	26.9 ± 4.5	28.1 ± 5.2	25.1 ± 5.1	27.8 ± (–)	6.137	0.105
AST (U/L)	27.1 ± 7.4	29.7 ± 9.0	28.6 ± 10.7	28.5 ± (–)	1.018	0.797
ALP(U/L)	80.0 ± 8.5	80.0 ± 7.0	80.3 ± 11.9	80.0 ± (–)	0.533	0.912
TBL(mg/dL)	0.83 ± 0.30	1.07 ± 0.33^**a, b**^	0.75 ± 0.20	0.65 ± (–)	**16.591**	**0.001**
DBL(mg/dL)	0.27 ± 0.16	0.30 ± 0.16	0.23 ± 0.14	0.40 ± (–)	4.345	0.227
Total protein (g/dL)	7.55 ± 0.63	7.40 ± 0.50	7.61 ± 0.75	7.26 ± (–)	1.837	0.607
Albumin (g/dL)	4.41 ± 0.66	4.30 ± 0.82	4.63 ± 0.18)	4.25 ± (–)	4.164	0.244
Globulin (g/dL)	3.08 ± 0.43	3.19 ± 0.65)	3.19 ± 0.67	2.91 ± (–)	0.524	0.914
A/G ratio	1.43 ± 0.38	1.40 ± 0.51)	1.45 ± 0.38	1.46 ± (–)	1.825	0.609
BChE(U/L)	5412 ± 1394	5178 ± 1278	5845 ± 1608	5764 ± (–)	5.572	0.134

Note:.

Interpretation for the irrigation group should be cautiously due to the small sample size (*n* = 3).

IQR was not calculated for the irrigation group due to small sample size (*n* = 3). Data are presented as median ± interquartile range (IQR). Independent-sample Kruskual-Wallis test was used to compare groups. Significant pairwise comparisons were determined using Bonferroni-adjusted post-hoc tests.

a- adjusted P. value < 0.05 when Packing compared with spraying.

b- adjusted P. value < 0.05 when packing compared with irrigation.

### Comparison of abnormal results of liver function and butyrylcholinesterase function tests among pesticide-exposed and controls

**Fig. 4:**
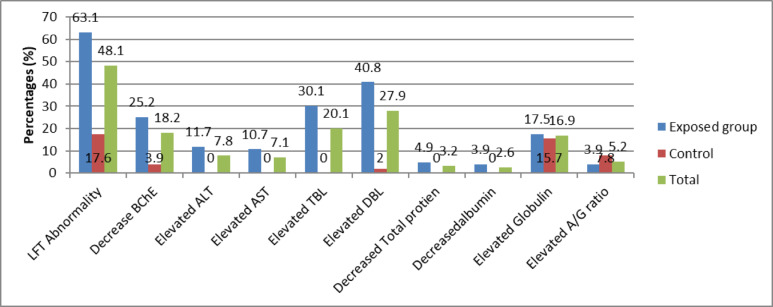
Comparison of abnormal LFT results and BChE among pesticide-exposed and controls. Abnormal LFTs and BChE levels were defined based on standard reference ranges. For LFTs, values above or below the normal reference ranges were considered abnormal. For BChE, values below the normal reference ranges were considered abnormal. Abnormal LFTs (*p* < 0.001) and decreased BChE (*p* < 0.001) were observed in the exposed group. Significant elevations were noted in ALT (*p* = 0.009), AST (*p* = 0.016), (TBL, *p* < 0.001), and DBL (*p* < 0.001). Non-significant changes were observed in total protein (*p* = 0.171), albumin (*p* = 0.302), globulin (*p* = 0.744), and A/G ratio (A/G, *p* = 0.441).

Pesticide-exposed individuals had significantly high LFT abnormality 65/103(63.1%) compared to controls 19/51(17.6%), *p* < 0.001. About 12/103(11.7%) of floriculture workers had elevated ALT (*p* = 0.009) and 11/103(10.7%) elevated AST (*p* = 0.016), while no abnormalities were observed in controls. Pesticide exposed participants had high TBL than controls 31/103(30.1%) vs. 0(0%), *p* < 0.001) and DBL 42/10 (40.8%) vs. 1(2%), *p* < 0.001). There were no significant differences between pesticide-exposed and controls in total proteins, albumin, globulin, and albumin to globulin ratio. A significantly higher proportion of exposed workers had low BChE levels compared to controls, 26/103(25.2%) vs.2/51 (3.9%), *p* = 0.001 (Fig. 4).

### Effect of work duration on liver function tests and serum cholinesterase in pesticide-exposed workers

A one-way ANOVA was conducted to compare the effect of work duration (< 5years, 5–10 years, and > 10 years) on BChE, LFT, and BMI. ALT levels showed a significant difference.

across groups, F (2, 100) = 11.798, *p* < 0.001. Tukey post hoc comparisons showed that participants worked > 10 years had significantly higher ALT levels (30.43 ± 3.30 U/L) than both 5–10 years (27.43 ± 3.28 U/L, p = < 0.001) and < 5 years (26.34 ± 2.46 U/L, *p* < 0.001), but the difference between < 5 and 5–10 years was not significant (*p* = 0.246). AST levels also revealed a significant difference with work duration, F (2, 100) = 12.022, *p* < 0.001. Post hoc analysis showed higher AST in > 10 years (33.35 ± 5.78 U/L) compared to 5–10 years (27.48 ± 4.66 U/L, *p* < 0.001) and < 5 years (26.92 ± 5.05 U/L, *p* < 0.001). The difference between < 5 and 5–10 years was not significant (*p* = 0.869) (Table 8). The details of one-way ANOVA are provided in supplementary information (Table S2).

**Table 8:** Effect of work duration on LFT and BChE level among pesticide-exposed participants (*N* = 103).

Test Parameter	Duration of Exposure in years			ANOVA F(*P*.value)	Linear contrast estimate	Eta squared (η²)
	< 5 years (mean ± SD)	5–10 years (mean ± SD)	> 10 years (mean ± SD)			
ALT (U/L)	26.34 ± 2.46	27.43 ± 3.28	30.43 ± 3.30	11.798(< 0.001)^**a, b**^	2.982	0.191
AST (U/L)	26.92 ± 5.05	27.48 ± 4.66	33.35 ± 5.78	12.022 (< 0.001)^**a, b**^	4.543	0.194
ALP (U/L)	79.09 ± 6.93	78.70 ± 6.36	80.06 ± 4.70	0.330(0.720)	0.689	0.007
TBL (mg/dL)	0.76 ± 0.18	0.85 ± 0.17	1.10 ± 0.20	23.484(< 0.001)^**a, b**^	0.241	0.320
DBL (mg/dL)	0.26 ± 0.089	0.25 ± 0.095	0.39 ± 0.10	14.405(< 0.001)^**a, b**^	0.090	0.247
Total Protein (mg/dL)	7.47 ± 0.44	7.55 ± 0.42	7.35 ± 0.56	1.288(0.280)	−0.081	0.025
Albumin (mg/dL)	4.50 ± 0.37	4.43 ± 0.40	3.97 ± 0.45	11.966(< 0.001)^**a, b**^	−0.370	0.193
Globulin (mg/dL)	2.97 ± 0.40	3.12 ± 0.42	3.38 ± 0.41	6.331(0.003)^**c, d**^	0.289	0.112
A/G ratio	1.55 ± 0.30	1.46 ± 0.31	1.19 ± 0.20	9.862(< 0.001)^**a, d**^	−0.251	0.165
BChE (U/L)	5538.60 ± 1124.95	5370.52 ± 842.13	4536.25 ± 842.26	7.607(< 0.001)^**c, d**^	−708.768	0.133
Total (N)	35	48	20			

Note: F-test (One-way ANOVA) with post hoc multiple comparisons is used to compare means.

• *p* < 0.001, > 10 years compared to < 5 years (One-way ANOVA/Tukey post hoc test).

• *P* < 0.001, > 10 years compared to 5–10 years (One-way ANOVA/Tukey post hoc test).

• *P* < 0.05, > 10 years compared to < 5years (One-way ANOVA/tukey post hoc test).

• *p* < 0.05, > 10 years compared to 5–10 years (One-way ANOVA/Tukey post hoc test).

• N- Number of study participants.

### Factors associated with serum cholinesterase and abnormal liver function tests

After adjusting for covariates in multivariate binary logistic regression analyses, PPE use, washing hands after work, and eating/drinking in the work area were significantly associated with abnormally low BChE. Workers who used PPE had 91% lower odds of having low BChE compared with those who did not (AOR = 0.09, 95% CI (0.01, 0.73), *p* = 0.024). Similarly, washing hands after work was associated with 81% lower odds of low BChE (AOR = 0.19, 95% CI (0.05, 0.75), *p* = 0.017). In contrast, workers who drank/ate in the work area had 5.91 times higher odds of having a low BChE level compared with those who did not (AOR = 5.91, 95% CI (1.50, 23.24), *p* = 0.011). Supplementary file 4 (Table S3). After adjustment for confounding variables, work duration greater than 10 years was significantly associated with higher odds of LFT abnormality (AOR = 7.75, 95% CI (1.45, 41.47), *p* = 0.017), compared with those working less than 5 years Supplementary file 4 (Table S4).

## Discussion

The present study aimed to assess the effect of pesticides on LFT and BChE levels among floriculture workers in Ethiopia and associated factors with LFT abnormality and abnormal BChE levels. Biochemical analysis of the study revealed altered levels of LFT and BChE among pesticide-exposed participants compared to controls. Lack of PPE use, no were hand washing, and eating/drinking in the working area were significantly associated with abnormally low BChE levels. Work duration greater than 10 years is significantly associated with abnormal LFT levels.

During the study, the most commonly reported symptoms among pesticide-exposed floriculture workers included excessive sweating, skin rash, cough, eye irritation, and headache. Different literatures reported similar findings^9,23,48–51^.

In the present study, about 34 chemicals were identified. According to WHO hazard classifications, 18 pesticides (53%) were Class U (unlikely to present acute hazard), 13 pesticides (38%) were Class II (moderately hazardous), 2 pesticides (6%) were Class Ib (highly hazardous), and 1 pesticide (3%) was Class III (slightly hazardous)^52^. 6 (17.6%) of the pesticides constitute organophosphates, and 3 (8.8%) are carbamates found in the pesticides in our study; these findings were supported by different studies^53–55^. Organophosphates and carbamates are known for their neurotoxic effects, inhibiting cholinesterase activity and potentially leading to acute poisoning or long-term neurological impairment^56^. In our study, participants were exposed to mixed pesticides. The combination of two or more chemicals can have synergistic effects, which may increase their toxicity and potentially harm various organ systems in the body^57^.

In the present study, floriculture workers had significantly altered levels of LFTs. These results are generally consistent with evidence from various occupational pesticide exposure studies, though some parameters differed across regions, pesticide types, and study populations. There were significant elevation of ALT (27.71 ± 0.44 U/L vs. 22.71 ± 0.47 U/L; P. value < 0.001) and AST (28.46 ± 0.68 U/L vs. 20.69 ± 0.73 U/L, P. value < 0.001) in pesticide exposed compared to control in our study, which is consistent with Thailand^20^, Pakistan and Cameron^58^, Cameron^59^, Iraq^21^, India^23^, Brazil^22^, Benin^49^, and Egypt^60^. However, in certain studies, ALT and AST did not significantly differ between pesticide-exposed and controls, Thailand^61^, and Iran^62^. Other studies, Cameron and Tanzania, showed that a significant increase in ALT but not AST in pesticide-exposed individuals compared to controls^63,64^. The discrepancy might be due to shorter exposure durations, different pesticide formulations, different study setups, and effective use of personal protective equipment.

Our study showed that no significant ALP (80.04 ± 0.94 U/L vs. 78.76 ± 1.00 U/L; P- value-0.351) difference between floriculture workers and controls, it lined with Thailand^61^, Pakistan, and Cameroon^58^. Our study contrasted with different studies conducted in Thailand^20^, Iraq^21^, India^23^, Cameroon^59^, and Egypt^60^. The variability in ALP findings might be related to pesticide class, with certain compounds more likely to induce cholestatic injury.

In this study, there was a significant increase in TBL (0.831 ± 0.028 mg/dL vs. 0.362 ± 0.030 mg/dL; P-value < 0.001) in pesticide-exposed participants than in controls. Our study aligned with a study conducted in Iraq^21^. The study contrasted with the study conducted in Cameron and Pakistan^58^. There was also a significant increase in DBL (0.298 ± 0.012 mg/dL vs. 0.170 ± 0.021 mg/dL; P-value < 0.001) in floriculture workers than in controls. It was consistent with a study conducted in India^23^. While it contrasted with studies conducted on Pakistan and Cameroon, and Brazil, with no significant difference between cases and controls^22,58^. Our study suggested that impaired bilirubin metabolism or excretion, potentially due to hepatocellular or canalicular dysfunction. The discrepancy might be due to differences in study setup, pesticide type, and intensity of exposure.

Our study revealed a significant reduction of total protein (7.385 ± 0.058 g/dL vs. 7.624 ± 0.098 g/dL; P-value − 0.038) in pesticide-exposed compared to controls, supported by a study conducted in India^23^, and Benin^49^. There were also a significant reduction of albumin (4.255 ± 0.052 g/dL vs. 4.623 ± 0.088 g/dL; P-value < 0.001) in pesticide-exposed compared to controls, in line with a study conducted in Thailand^61^. In contrast to our study, the study from India^23^ reported increased albumin in exposed workers, potentially reflecting differences in nutritional status, hydration. Moreover, a significant decrease in A/G ratio (1.394 ± 0.040 vs. 1.580 ± 0.067; P-value- 0.018) was observed pesticide-exposed than controls in our study, which was parallel with^65^. Our study indicated that reduced hepatic synthetic capacity occurs under chronic pesticide exposure.

In this study, significantly lower BChE was observed in cases (5105 ± 141U/L vs. 6699 ± 239U/L; P-value < 0.001) compared with controls. It was due to the presence of OPs and CMs pesticides in our study. Organophosphates and carbamates are commonly used pesticides on flower farms in Ethiopia^55,66,67^. Our study agreed with different studies in Thailand^61^, Iraq^21^, Brazil^22^, and Cameroon^59^.

In our study, 26/103 (25.2%) of floriculture workers had abnormally low BChE levels. It was consistent with findings from occupational pesticide-exposed workers in Iran, which was 23%^62^. Our study was lower than the study conducted in Thailand with abnormal Low BChE (46.5%)^68^. The prevalence was higher among male participants (29.7%) compared to females (22.7%), indicating potential chronic exposure to cholinesterase-inhibiting pesticides, specifically OPs (17.6%) and CMs (8.8%), in our study area. In our investigation, the sex-specific difference that was observed Males are more likely than females to have abnormally low BChE, which could be caused by variations in work tasks, exposure levels, and duration. In many floriculture farms, female workers are usually assigned to post-harvest or greenhouse duties with relatively lower direct exposure, whereas male workers are more likely to be involved in pesticide mixing, spraying, and application, which include higher cutaneous and inhalation risks^66^.

The present study demonstrated a significant reduction in BChE among floriculture workers with more than 10 years of pesticide exposure compared to those with shorter durations (less than 5 years and 5–10 years). This finding suggests a cumulative inhibitory effect of long-term exposure to cholinesterase-inhibiting pesticides, primarily OPs and CMs, which are commonly used in the floriculture industry^69^.

In our investigation, workers with more than 10 years of exposure had significantly higher ALT, AST, TBL, and DBL levels compared to both the less than 5 years and 5–10 years exposure groups. In contrast, albumin and the A/G ratio showed a significant decrease in workers with more than 10 years of exposure compared to the other two groups. These findings indicated progressive hepatocellular injury associated with chronic pesticide exposure. Prolonged exposure to Ops and CMs pesticides could induce oxidative stress, mitochondrial damage, and disruption of hepatocellular membranes, leading to the leakage of transaminases into the bloodstream^70^.

Workers who used PPE in the work area had lower odds of having low BChE compared with those who did not (AOR = 0.09, 95% CI: 0.01, 0.73), *p* = 0.024). This finding was supported by evidence from a study conducted in Brazil, which highlighted the protective role of PPE against cholinesterase inhibition among agricultural workers^71^. Those finding study supported by the study conducted Pakistan tobacco workers who did not wear PPE had higher odds of having abnormal BChE^72^. Similarly, washing hands after work was associated with lower odds of low BChE (AOR = 0.19, 95% CI: 0.05, 0.75), *p* = 0.017), in line with the study done in Pakistani tobacco workers^72^. In contrast, workers who drank/ate in the work area had higher odds of low BChE level compared with those who did not (AOR = 5.91, 95% CI (1.50, 23.24). Work duration greater than 10 years was significantly associated with higher odds of LFT abnormality (AOR = 7.75, 95% CI (1.45, 41.47), *p* = 0.017), compared with those working less than 5 years. The long-term exposure to pesticides is known to exert adverse effects on total proteins, albumin, ALT, AST, and TBL, resulting in hepatic toxicity^73^.

### Strengths and limitations of the study

One of the strengths of this study was that the participants were selected independently, without interference from farm administrators, which enhanced the validity of the findings. The other strength of the study was that all laboratory analyses were performed at the EPHI Clinical Chemistry Reference Laboratory, the national reference facility, ensuring the reliability and validity of results. Moreover, this is the first biochemical study conducted in Ethiopia, and to our knowledge, the first in East Africa, focusing on workers in the floriculture industry. Additionally, the use of a comparative cross-sectional design, coupled with extensive statistical analyses, including Independent t-test, ANOVA, ANCOVA, Chi-square, Bivariate and multivariate logistic regression, further strengthened the validity and robustness of the study outcomes.

There are some limitations to this study. The study cannot demonstrate a clear cause-and-effect relationship between pesticide exposure and the health effects, since research employed a cross-sectional methodology. Recall bias may have been generated since some of the data like reported symptoms and pesticide use habits were dependent on participants’ memories. HBV and HCV serological screening was not performed in this study. As viral hepatitis is a well-established cause of elevated aminotransferase levels, the absence of screening may have introduced residual confounding. Consequently, some of the observed alterations in ALT and AST levels may not be solely attributable to pesticide exposure. Additionally, because the workers had already been exposed to pesticides regularly, we were unable to get baseline LFTs and BChE levels before their employment. Furthermore, we did not gather comprehensive information on the precise quantities of pesticides applied, the frequency of spraying, or the time after which workers returned to the greenhouse after spraying. Finally, the lack of similar studies in Ethiopia and East Africa made it difficult to compare our results with those from other populations.

## Conclusion and recommendations

The study also revealed that significant increases in levels of LFTs, including ALT, AST, DBL, and TBL compared to controls in floriculture workers. Conversely, significantly low levels of BChE, Total protein, Albumin, and A/G ratio in floriculture compared to controls, indicating hepatotoxicity of pesticides. About 25.2% of the pesticide-exposed participants had abnormally low BChE levels caused by exposure to high levels of organophosphates and carbamates. The LFTs, including ALT, AST, TBL, DBL, and globulin, were significantly increased with the duration of exposure to pesticides in participants. In contrast, albumin and the A/G ratio were significantly decreased with increased work duration. It showed that the increased progressive hepatotoxicity of pesticides increases with duration. Pesticides exposed to pesticides for longer had a significant association with LFT abnormality. Use of PPE, hand washing habit, and habit of eating/drinking in the workplace had a significant association with abnormal BChE level.

Floriculture industry owners are encouraged to provide full PPE to floriculture workers. Floriculture industry owners are recommended to provide periodical check-ups to floriculture workers for early recognition of health problems. Training on safe pesticide use and the potential adverse effects of pesticides should be provided to all workers. The responsible health sectors should actively monitor the health status of floriculture workers and raise awareness regarding pesticide toxicity. Workers are also advised to consistently take protective measures at the workplace, including proper use of PPE. Further research is recommended, particularly longitudinal studies across multiple floriculture sites, to develop a comprehensive understanding of occupational pesticide exposure in Ethiopia.

## Electronic Supplementary Material

Below is the link to the electronic supplementary material.

Supplementary Material 1

Supplementary Material 2

Supplementary Material 3

Supplementary Material 4

## Data Availability

The data sets used and/or analyzed during the current study are available from the corresponding author on reasonable request.

## Abbreviations

ACHE: Acetylcholine esterase
A/G ratio: Albumin to globulin ratio
ALP: Alkaline phosphatase
ALT: Alanine aminotransferase
ANCOVA: Analysis of Covariance ANOVA: Analysis of variance
API: Acute pesticide intoxication
AST: Aspartate aminotransferase
BChE: Butyrylcholinesterase
CM: Carbamates
CSA: Central Statistical Agency
DBL: Direct Bilirubin
EPHI: Ethiopian Public Health Institute
EU: European Union
GLM: General linear model
IFCC: International Federation of Clinical Chemistry
IQC: Internal quality control
LFTs: Liver function test
MD: Mean difference OP: Organophosphate
PPE: Personal protective equipment
ROS: Reactive oxygen specious
SE: Standard error
SOP: Standard operating procedure
SPSS: Software package for social science
SST: Serum separator tube
UAPP: Unintentional Acute Pesticide Poisoning: UNFAO: United Nations Food and Agriculture Organization
USD: United States dollar
WHO: World Health Organization
