# Supplementary material for: Effect of pesticide exposure on liver function tests and serum cholinesterase levels among floriculture industry workers in Bahirdar, Ethiopia: a comparative cross-sectional study
Source: Sci Rep. 2026 May 3;16:21885. doi: 10.1038/s41598-026-51363-8 (PMC13365533; doi:10.1038/s41598-026-51363-8)
Supplement: Supplementary file 1 — Supplementary Material 1 [file 41598_2026_51363_MOESM1_ESM.docx]

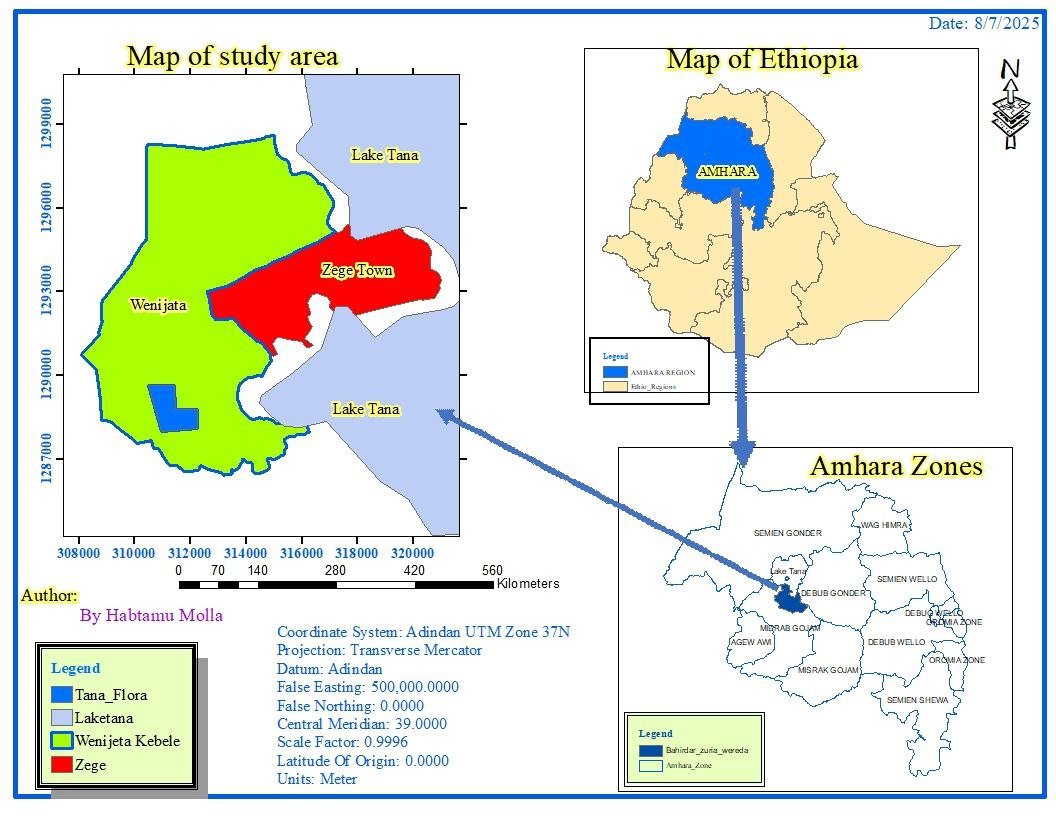


Figure S1: Map of the study area

**Sampling procedures**

A floriculture farm includes a majority of 4 areas: green
houses, pack houses, irrigation, and pesticide spraying. Building flower beds, applying fertilizers and pesticides, planting, working in flower beds, weeding and cutting, gathering flowers, raising flowerbeds, pruning, and transporting organic waste are all done in the greenhouse part.
Employees in the greenhouse are more likely to be exposed to pesticides because they work all day in an enclosed area with a lot of chemicals. In the spraying department, pesticides are typically mixed and manually sprayed using spray lances. Walking into the spray mist while manually spraying with spray lances increases exposure to pesticides through the skin and respiratory systems (1).

The pack house is where post-harvest operations take place, and gathered flowers are arranged for export. It's likely that workers in the pack house were exposed to high pesticide doses while carrying out their tasks in an enclosed space to preserve flower quality. The irrigation section was in responsible for monitoring water lines and mixing fertilizers and other substances required for flower growth. Workers in this department combine different fertilizer ingredients (2).

A proportional simple random and stratified sampling was employed in the study to select the cases. The floriculture industry had a total of 591 (215 Male and 376 Female) active workers. 37 (25 male, 12 Female) workers were administrative and managers and excluded from the studies. 64 (24 Male, and 40 Female) workers were labor workers temporary employed workers who worked less than 6 months and were excluded from the study. The remaining 490 workers were included in the study. From 490, 320 workers were from Greenhouse (flower cutting) (232 Females: 88 Males), 126 were from Packing (89 Females: 37 Males), 33 from the spray (all Males), and 11 from irrigation (all Male). 105 participants were recruited for the study. 2 participants from Greenhouse had known liver disease and were excluded from the study. Proportionally, 27(8 Male: 19 Female) from packing house, 65 (47 Females: 18 Males) from greenhouse, 8 from spray (all Males), 3 from irrigation (all Males) were included. The final sample size was 103: 37 Male and 66 Female. Controls were selected using a purposive sampling technique from households in Zege town across three kebeles (the smallest administrative units of the government). A total of 18 households were selected from Kebele 1, 17 from Kebele 2, and 17 from Kebele 3 using simple random sampling. From each selected household, one participant was purposively recruited and matched with a case by age and sex. Initially, 52 controls (34 females and 18 males) were enrolled in the study. However, one female participant with a known history of liver disease was excluded. Consequently, the final control group consisted of 51 participants (33 females and 18 males) (Figure S2).


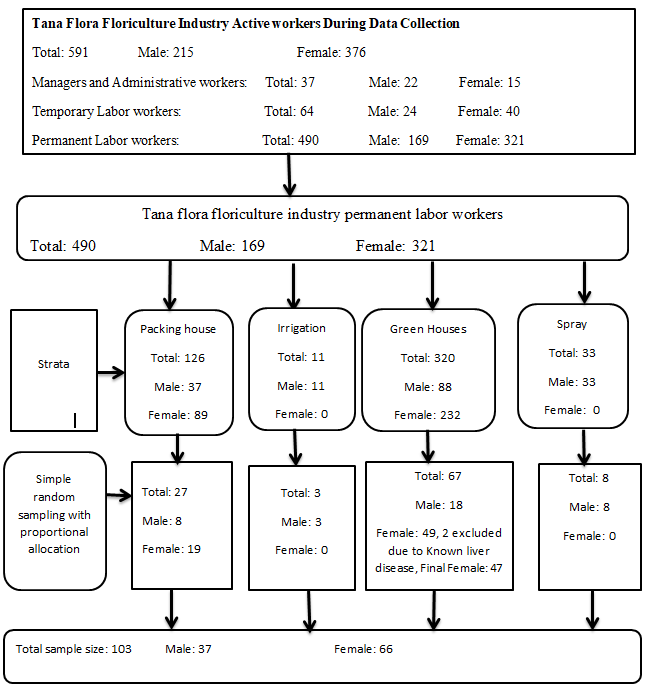


Figure S2: Sampling procedure of assessment of liver and renal function tests among horticultural workers at Tana flora floriculture industry, Bahirdar, Ethiopia, 2025.
