# Supplementary material for: Effect of pesticide exposure on liver function tests and serum cholinesterase levels among floriculture industry workers in Bahirdar, Ethiopia: a comparative cross-sectional study
Source: Sci Rep. 2026 May 3;16:21885. doi: 10.1038/s41598-026-51363-8 (PMC13365533; doi:10.1038/s41598-026-51363-8)
Supplement: Supplementary file 2 — Supplementary Material 2 [file 41598_2026_51363_MOESM2_ESM.docx]

**Analysis of Covariance for Liver function tests and serum cholinesterase**

Analysis of covariance (ANCOVA) was performed to examine differences in liver function tests LFTs) and serum cholinesterase (BChE) between pesticide-exposed workers and controls, while adjusting for sex, age, income, and residency. Pesticide exposure was significantly associated with altered LFTs and BChE, characterized by increase ALT,AST, TBL,DBL, alongside reductions in total protein, albumin, A/G ratio, BChE. In contrast, ALP and globulin levels were not significantly affected. Covariates, including age, sex, and income, also showed significant associations with selected outcomes.

Pesticide exposure was significantly associated with higher ALT, F(1) = 32.15, p < .001, and AST, F(1) = 26.46, p < .001. No significant difference was observed for ALP between exposed and control groups, F(1) = 0.29, p = .592. A significant age * exposure interaction was detected for ALT, F(1) = 4.57, p = .034, indicating that the effect of pesticide exposure on ALT levels varied across age groups.

Pesticide exposure was also significantly associated with elevated TBL, F(1) = 57.85, p < .001, and DBL, F(1) = 27.97, p < .001. Age showed a significant main effect on both TBL, F(1) = 5.36, p = .022, and DBL, F(1) = 7.93, p = .006. In addition, a significant income * exposure interaction was observed for TBL, F(2) = 3.54, p = .032, and DBL, F(2) = 3.22, p = .043, suggesting that the relationship between pesticide exposure and bilirubin levels differed across income categories.

Pesticide exposure was associated with lower albumin levels, F(1, 142) = 13.10, p < .001, total protein, F(1, 142) = 4.37, p = .038, A/G ratio, F(1) = 5.71, p = .018 and BChE , F(1) = 32.99, p < .001. In contrast, globulin levels did not differ significantly between groups, F(1) = 1.33, p = .250. Income had a significant effect on albumin, F(2) = 5.92, p = .003, and A/G ratio, F(2) = 4.76, p = .010. Additionally, sex was significantly associated with BChE levels, F(1) = 4.16, p = .043 (Table S1).

Table S1: Effects of pesticide exposure, age, sex, income and residency on LFTs and Serum Cholinesterase.

| **Variable** | **Effect** | **df** | **F** | **p-value** |
| --- | --- | --- | --- | --- |
| ALT (U/L) | Exposure | 1 | **32.15** | **<0.001** |
|  | Sex | 1 | 0.92 | 0.339 |
|  | Age | 1 | 1.39 | 0.241 |
|  | Income | 2 | 0.29 | 0.748 |
|  | Residency | 1 | 0.46 | 0.500 |
|  | Sex * Exposure | 1 | 0.01 | 0.927 |
|  | Age * Exposure | 1 | **4.57** | **0.034** |
|  | Income * Exposure | 2 | 0.49 | 0.614 |
|  | Residency * Exposure | 1 | 0.04 | 0.850 |
| AST (U/L) | Exposure | 1 | **26.46** | **<0.001** |
|  | Sex | 1 | 0.09 | 0.760 |
|  | Age | 1 | 3.83 | 0.053 |
|  | Income | 2 | 0.93 | 0.397 |
|  | Residency | 1 | 3.59 | 0.060 |
|  | Sex * Exposure | 1 | 1.92 | 0.168 |
|  | Age * Exposure | 1 | 0.50 | 0.483 |
|  | Income * Exposure | 2 | 2.00 | 0.140 |
|  | Residency * Exposure | 1 | 0.28 | 0.601 |
| ALP (U/L) | Exposure | 1 | 0.288 | 0.592 |
|  | Sex | 1 | 0.356 | 0.552 |
|  | Age | 1 | 1.150 | 0.286 |
|  | Income | 2 | 0.300 | 0.741 |
|  | Residency | 1 | 0.448 | 0.505 |
|  | Sex * Exposure | 1 | 0.009 | 0.925 |
|  | Age * Exposure | 1 | 1.551 | 0.215 |
|  | Income * Exposure | 2 | 0.823 | 0.442 |
|  | Residency * Exposure | 1 | 0.062 | 0.805 |
| TBL (mg/dl) | Exposure | 1 | **57.845** | **<0.001** |
|  | Sex | 1 | 0.140 | 0.708 |
|  | Age | 1 | **5.358** | **0.022** |
|  | Income | 2 | 1.476 | 0.233 |
|  | Residency | 1 | 0.708 | 0.402 |
|  | Sex * Exposure | 1 | 1.840 | 0.177 |
|  | Age * Exposure | 1 | 0.003 | 0.957 |
|  | Income * Exposure | 2 | **3.538** | **0.032** |
|  | Residency * Exposure | 1 | 2.934 | 0.089 |
| DBL (mg/dl) | Exposure | 1 | **27.968** | **<0.001** |
|  | Sex | 1 | 0.012 | 0.913 |
|  | Age | 1 | **7.925** | **0.006** |
|  | Income | 2 | 0.420 | 0.658 |
|  | Residency | 1 | 0.256 | 0.614 |
|  | Sex * Exposure | 1 | 0.082 | 0.776 |
|  | Age * Exposure | 1 | 0.029 | 0.866 |
|  | Income * Exposure | 2 | **3.219** | **0.043** |
|  | Residency * Exposure | 1 | 0.830 | 0.364 |
| Total Protein (g/dl) | Exposure | 1 | **4.367** | **0.038** |
|  | Sex | 1 | 1.157 | 0.284 |
|  | Age | 1 | 0.019 | 0.890 |
|  | Income | 2 | 0.404 | 0.669 |
|  | Residency | 1 | 0.095 | 0.758 |
|  | Sex * Exposure | 1 | 1.909 | 0.169 |
|  | Age * Exposure | 1 | 3.571 | 0.061 |
|  | Income * Exposure | 2 | 1.248 | 0.290 |
|  | Residency * Exposure | 1 | 2.971 | 0.087 |
| Albumin (g/dl) | Exposure | **1** | **13.101** | **<0.001** |
|  | Sex | 1 | 2.572 | 0.111 |
|  | Age | 1 | 2.552 | 0.112 |
|  | Income | 2 | **5.916** | **0.003** |
|  | Residency | 1 | 0.724 | 0.396 |
|  | Sex * Exposure | 1 | 0.144 | 0.705 |
|  | Age * Exposure | 1 | 2.953 | 0.088 |
|  | Income * Exposure | 2 | 2.683 | 0.072 |
|  | Residency * Exposure | 1 | 0.016 | 0.898 |
| Globulin (g/dl) | Exposure | 1 | 1.333 | 0.250 |
|  | Sex | 1 | 0.130 | 0.719 |
|  | Age | 1 | 1.701 | 0.194 |
|  | Income | 2 | 2.421 | 0.092 |
|  | Residency | 1 | 0.209 | 0.649 |
|  | Sex * Exposure | 1 | 3.038 | 0.083 |
|  | Age * Exposure | 1 | 0.130 | 0.719 |
|  | Income * Exposure | 2 | 1.149 | 0.320 |
|  | Residency * Exposure | 1 | 3.467 | 0.065 |
| A/G Ratio | Exposure | 1 | **5.713** | **0.018** |
|  | Sex | 1 | 0.592 | 0.443 |
|  | Age | 1 | 2.173 | 0.143 |
|  | Income | 2 | **4.762** | **0.010** |
|  | Residency | 1 | 0.338 | 0.562 |
|  | Sex * Exposure | 1 | 2.086 | 0.151 |
|  | Age * Exposure | 1 | 0.313 | 0.577 |
|  | Income * Exposure | 2 | 1.895 | 0.154 |
|  | Residency * Exposure | 1 | 2.404 | 0.123 |
| BChE (U/L) | Group | 1 | **32.988** | **<0.001** |
|  | Sex | 1 | 4.159 | 0.043 |
|  | Age | 1 | 0.283 | 0.596 |
|  | Income | 2 | 2.407 | 0.094 |
|  | Residency | 1 | 0.160 | 0.690 |
|  | Group * Sex | 1 | 1.293 | 0.257 |
|  | Group * Age | 1 | 2.874 | 0.092 |
|  | Group * Income | 2 | 0.790 | 0.456 |
|  | Group * Residency | 1 | 0.894 | 0.346 |

Table S2: The effect of duration of exposure on liver function and Butrylcholinesterase in pesticide-exposed workers at Tana Flora Floriculture industry, Bahir Dar, Ethiopia, 2025(N=103)

| Study groups | Descriptives | ANOVA results | Levene's statistics test of homogeneity of variance based on the mean  (F(df1,df2)=F-statistic, P-value) | Tukey HSD Post hoc analysis | |
| --- | --- | --- | --- | --- | --- |
|  | **Variables**  **(mean± SD)** | **F(P.value)** |  | **Paired groups** | **P.value** |
|  | **ALT (U/L)** |  |  |  |  |
| < 5 years | 26.34 ± 2.46 | 11.798( <0.001) | F(2,100) = 2.010, p. value = 0.139 | <5 < years vs.5-10 years | 0.246 |
| 5-10 years | 27.43 ± 3.28 |  |  | 5-10 years vs.>10 years | **<0.001** |
| >10 years | 30.43 ± 3.30 |  |  | >10 years vs. <5 years | **<0.001** |
|  | **AST(U/L)** |  |  |  |  |
| < 5 years | 26.92 ± 5.05 | 12.022 (<0.001) | F(2,100)=0.741,p.value -0.479 | <5 years vs.5-10 years | 0.869 |
| 5-10 years | 27.48 ± 4.66 |  |  | 5-10 years vs.>10 years | **<0.001** |
| >10 years | 33.35 ± 5.78 |  |  | >10 years vs. <5 years | **<0.001** |
|  | **ALP(U/L)** |  |  |  |  |
| <5 years | 79.09 ± 6.93 | 0.330(0.720) | F(2,100)=0.947,p.value -0.391 | <5 years vs.5-10 years | 0.956 |
| 5-10 years | 78.70 ± 6.36 |  |  | 5-10 years vs. > 10 years | 0.696 |
| >10 years | 80.06 ± 4.70 |  |  | >10 years vs. < 5 years | 0.845 |
|  | **TBL(mg/dl)** |  |  |  |  |
| < 5 years | 0.76 ± 0.18 | 23.484(<0.001) | F(2,100)=0.265, P. P-value -0.768 | <5 years vs.5-10 years | 0.072 |
| 5-10 years | 0.85 ± 0.17 |  |  | 5-10 years vs.>10 years | **<0.001** |
| >10 years | - 1. ± 0.20 |  |  | >10 years vs. <5 years | **<0.001** |
|  | **DBL(mg/dl)** |  |  |  |  |
| < 5 years | 0.26 ± 0.089 | 14.405(<0.001) | F(2,100)=0.04, p. value-0.952 | <5 years vs.5-10 years | 0.868 |
| 5-10 years | 0.25 ±0.095 |  |  | 5-10 years vs.>10 years | **<0.001** |
| >10 years | 0.39± 0.10 |  |  | >10 years vs. <5 years | **<0.001** |
|  | **Total protein (mg/dl)** |  |  |  |  |
| 2-6 years | 7.47 ± 0.44 | 1.288(0.280) | F(2,100) =1.631, p. value-0.20 | <5 years vs.5-10 years | 0.722 |
| 7-10 years | 7.55 ± 0.42 |  |  | 5-10 years vs.>10 years | 0.255 |
| >10 years | 7.35 ± 0.56 |  |  | >10 years vs. <5 years | 0.643 |
|  | **Albumin (mg/dl)** |  |  |  |  |
| < 5 years | 4.50 ± 0.37 | 11.966(<0.001) | F(2,100)=0.550, p. value-0.579 | <5 years vs.5-10 years | 0.714 |
| 5-10 years | 4.43 ± 0.40 |  |  | 5-10 years vs.>10 years | **<0.001** |
| >10 years | 3.97 ± 0.45 |  |  | >10 years vs. <5 years | **<0.001** |
|  | **Globulin(mg/dl)** |  |  |  |  |
| < 5 years | 2.97 ± 0.40 | 6.331(0.003) | F(2,100)=0.213, p.value-0.809 | <5 years vs.5-10 years | 0.242 |
| 5-10 years | 3.12 ± 0.42 |  |  | 5-10 years vs.>10 years | **0.048** |
| >10 years | 3.38 ± 0.41 |  |  | >10 years vs. <5 years | **0.002** |
|  | **Albumin to globulin ratio** |  |  |  |  |
| < 5 years | 1.55 ± 0.30 | 9.862(<0.001) | F(2,100)=1.064, p. value-0.349 | <5 years vs.5-10 years | 0.328 |
| 5-10 years | 1.46 ± 0.31 |  |  | 5-10 years vs.>10 years | **0.003** |
| >10 years | 1.19 ± 0.20 |  |  | >10 years vs. <5 years | **<0.001** |
|  | **BChE (U/L)** |  |  |  |  |
| < 5 years | 5538.60 ± 1124.95 | 7.607(<0.001) | F(2,100)=1.054, p. value = 0.359 | <5 years vs.5-10 years | 0.738 |
| 5-10 years | 5370.52 ± 842.13 |  |  | 5-10 years vs.>10 years | **0.002** |
| >10 years | 4536.25± 842.26 |  |  | >10 years vs. <5 years | **0.001** |
| Notes: P-value ≤ 0.05 is considered significant | | | | | |
