# Supplementary material for: Effect of pesticide exposure on liver function tests and serum cholinesterase levels among floriculture industry workers in Bahirdar, Ethiopia: a comparative cross-sectional study
Source: Sci Rep. 2026 May 3;16:21885. doi: 10.1038/s41598-026-51363-8 (PMC13365533; doi:10.1038/s41598-026-51363-8)
Supplement: Supplementary file 3 — Supplementary Material 3 [file 41598_2026_51363_MOESM3_ESM.docx]

| **Variables** | **Category** | **BChE level** | | **COR (95%Cl)** | **P.value** | **AOR (95%Cl)** | **P.value** |
| --- | --- | --- | --- | --- | --- | --- | --- |
|  |  | **Low n (%)** | **Normal n (%)** |  |  |  |  |
| Sex | Male | 11(29.7%) | 26(70.3%) | 1 | - | - | - |
|  | Female | 15(22.7%) | 51(77.3%) | 0.695(0.280, 1.727) | 0.434 | - | - |
| Age | 18-25 year | 11(17.5%) | 52(82.5%) | 1 | - | - | - |
|  | >25 year | 15(37.5%) | 25(62.5%) | 2.836(1.139,7.065) | 0.025* | 2.093(0.516, 8.483) | 0.301 |
| Residency | Rural | 19(23.2%) | 63(76.8%) | 1 | - | - | - |
|  | Urban | 7(33.3%) | 14(66.7%) | 1.658(0.585, 4.701) | 0.342 | - | - |
| Work duration | <5 years | 8(22.9%) | 27(77.1%) | 1 | - | - | - |
|  | 5-10 years | 9(18.8%) | 39(81.2%) | 0.779(0.267, 2.274) | 0.647 | 0.337(0.073, 1.550) | 0.162 |
|  | >10 years | 9(45%) | 11(55%) | 2.761(0.846, 9.010) | 0.092* | 1.474(0.245, 8.872) | 0.672 |
| BMI | Underweight | 2(25%) | 6(75%) | 1 | - |  |  |
|  | Normal | 21(23.1%) | 70(76.9%) | 0.90(0.169, 4.795) | 0.902 | 1.372(0.104, 18.183) | 0.810 |
|  | Overweight | 3(75%) | 1(25%) | 9.00(0.563, 143.880 | 0.120* | 3.649(0.097,137.544) | 0.485 |
| PPE use | No | 24(37.5%) | 40(62.5%) | 1 | - | - | - |
|  | Yes | 2(5.1%) | 37(94.9%) | 0.090(0.020, 0.408) | 0.002* | 0.091(0.011, 0.725) | **0.024** |
| Taking bath after work | No | 25(28.7%) | 62(71.3%) | 1 | - | - | - |
|  | Yes | 1(6.3%) | 15(93.8%) | 0.165 (0.021, 1.319) | 0.089* | 0.399(0.031, 5.134) | 0.481 |
| Washing hands after work | No | 12(52.2%) | 11(47.8%) | 1 | - | - | - |
|  | Yes | 14(17.5%) | 66(82.5%) | 0.194(0.071, 0.529) | 0.001* | 0.190 (0.049, 0.747) | **0.017** |
| Changing clothes after work | No | 21(34.4%) | 40(65.6%) | 1 | - | - | - |
|  | Yes | 5(11.9%) | 37(88.1%) | 0.257(0.088, 0.753) | 0.013* | 0.888(0.195, 4.032) | 0.877 |
| Drinking or eating in work area | No | 13(18.1%) | 59(81.9%) | 1 | - | - | - |
|  | Yes | 13(41.9%) | 18(58.1%) | 3.278(1.290, 8.328) | 0.013* | 5.908(1.502, 23.244) | **0.011** |
| Consumption of coffee | No | 10(22.7%) | 34(77.3%) | 1 | - | - | - |
|  | Yes | 16(27.1%) | 43(72.9%) | 1.265(0.510, 3.140) | 0.612 | - | - |
| Alcohol consumption | No | 18(23.1%) | 60(76.9%) | 1 | - | - | - |
|  | Yes | 8(32%) | 17(68%) | 1.569(0.582, 4.229) | 0.374 | - | - |
| Taking periodical check up | No | 24(30.4%) | 55(69.6%) | 1 | - | - | - |
|  | Yes | 2(8.3%) | 22(91.7%) | 0.208(0.045, 0.957) | 0.044* | 0.172(0.024,1.232) | 0.080 |
| Note: BChE- Butrylcholinesterase, PPE- Personal protective equipment’s, 1- Reference groups , *- Candidate variable for multivariate logistic regression model, Hosmer and Lemeshow test(χ²= 4.023, P. value= 0.855) | | | | | | | |

Table S2: Bivariate and multivariate logistic regression of factors associated with LF abnormality

Table S3: Bivariate and multivariate logistic regression of factors associated with LFT abnormality

| **Variables** | **Category** | **LFT Abnormality** | | **COR (95%Cl)** | **P.value** | **AOR (95%Cl)** | **P.value** |
| --- | --- | --- | --- | --- | --- | --- | --- |
|  |  | **Normal (%)** | **Abnormal n (%)** |  |  |  |  |
| Sex | Male | 19(51.4%) | 18(48.6%) | 1 | - | - | - |
|  | Female | 19(28.8%) | 47(71.2%) | 2.611(1.131,6.026 | 0.021* | 2.154(0.822,5.642) | 0.118 |
| Age | 18-25 year | 26(41.3%) | 37(58.7%) | 1 | - | - | - |
|  | >25 year | 12(30%) | 28(70%) | 1.640(0.706, 3.805) | 0.250 | - | - |
| Residency | Rural | 28(34.1%) | 54(65.9) | 1 | - | - | - |
|  | Urban | 10(47.6%) | 11(52.4%) | 0.570(0.216, 1.505) | 0.257 | - | - |
| BMI | Under weight | 12(70.6%) | 5(29.4%) | 1 | - | - | - |
|  | Normal | 67(50.4%) | 66(49.6%) | 2.364(0.789,7.083) | 0.124 | 1.655(0.292, 9.397) | 0.569 |
|  | Over weight | 1(25%) | 3(75%) | 7.200(0.596, 87.020) | 0.121* | 1.970(0.085, 45.641) | 0.672 |
| Work duration | <5 years | 17(48.6) | 18(51.4%) | 1 | - | - | - |
|  | 5-10 years | 19(39.6%) | 29(60.4%) | 1.442(0.598, 3.475) | 0.415 | 1.454(0.546, 3.871) | 0.453 |
|  | >10 years | 2(10%) | 18(90%) | 8.5(1.709, 42.279) | 0.009* | 7.745(1.447, 41.468) | **0.017** |
| PPE use | No | 24(37.5%) | 40(62.5%) | 1 | - | - | - |
|  | Yes | 14(35.9%) | 25(64.1%) | 1.071(0.469,2.450) | 0.870 | - | - |
| Taking bath after work | No | 31(36%) | 55(64%) | 1 | - | - | - |
|  | Yes | 7(42.2%) | 10(58.8%) | 0.805(0.279, 2.327) | 0.689 | - | - |
| Washing hands after work | No | 6(26.1%) | 17(73.9%) | 1 | - | - | - |
|  | Yes | 32(40%) | 48(60%) | 0.529(0.189,1.487) | 0.227* | 0.448(0.135, 1.483) | 0.189 |
| Changing clothes after work | No | 24(39.3%) | 37(60.7%) | 1 | - | - | - |
|  | Yes | 14(33.3%) | 28(66.7%) | 1.297(0.570,2.951) | 0.535 | - | - |
| Drinking or eating in work area | No | 23(31.9%) | 49(68.1%) | 1 | - | - | - |
|  | Yes | 15(48.9%) | 16(51.6%) | 0.501(0.212,1.185) | 0.115* | 0.543(0.201, 1.465) | 0.228 |
| Consumption of coffee | No | 14(31.8%) | 30(68.2%) | 1 | - | - | - |
|  | Yes | 24(40.7%) | 35(59.5%) | 0.681(0.300, 1.545) | 0.358 | - | - |
| Alcohol consumption | No | 29(37.2%) | 49(62.8%) | 1 | - | - | - |
|  | Yes | 9(36%) | 16(64%) | 1.052(0.412, 2.685) | 0.915 | - | - |
| Taking periodical check up | No | 28(35.4%) | 51(64.6% | 1 | - | - | - |
|  | Yes | 10(41.7%) | 14(58.3%) | 0.769(0.302, 1.955) | 0.581 |  |  |
| Note: LFT- Liver function tests, PPE- Personal protective equipment’s, 1- Reference groups , *- Candidate variable for multivariate logistic regression model, Hosmer and Lemeshow test(χ²= 7.025, P. value= 0.534 | | | | | | | |
