# Supplementary material for: Effect of pesticide exposure on liver function tests and serum cholinesterase levels among floriculture industry workers in Bahirdar, Ethiopia: a comparative cross-sectional study
Source: Sci Rep. 2026 May 3;16:21885. doi: 10.1038/s41598-026-51363-8 (PMC13365533; doi:10.1038/s41598-026-51363-8)
Supplement: Supplementary file 4 — Supplementary Material 4 [file 41598_2026_51363_MOESM4_ESM.docx]

**Principles of the laboratory tests**

1. **Liver function tests (LFTs)**
   1. Alanine aminotransferase (ALT)

Test Kit name: ALTL

Purpose

Numerous tissues have been found to contain the ALT. Because the liver is the primary source of ALT, ALT activity is measured to diagnose hepatic disorders. Hepatitis, cirrhosis, obstructive jaundice, liver cancer, and long-term alcohol misuse are all associated with elevated serum ALT levels. Patients with a simple myocardial infarction have ALT that is just marginally increased (1).

Principles

The principle of ALT measurement is based on the International Federation of Clinical Chemistry (IFCC) without the pyridoxal phosphate method. Alpha-ketoglutarate and L-alanine react to produce L-glutamate and pyruvate when ALT catalyzes the reaction. Pyruvate changes to lactate and NADH to NAD when LDH is present. The serum activity of ALT is directly proportional to the decrease in NADH absorbance, as measured at 340 nm. The reaction has a kinetic rate (2).


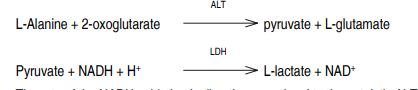


- 1. Aspartate aminotransferase (AST)

Test kit name: ASTL

Purpose

Aspartate aminotransferase, or AST, is a widely distributed enzyme in tissue, primarily in the kidney, heart, liver, and muscle. Diseases affecting these tissues are associated with elevated serum levels. Serum AST levels are also raised by hepatobiliary conditions, including viral hepatitis, metastatic cancer, and cirrhosis. Serum AST rises after myocardial infarction and peaks two days after the event (1).

The principle of AST measurement is based on the International Federation of Clinical Chemistry (IFCC) without the pyridoxal phosphate method. L-glutamate and oxaloacetate are produced when alpha-ketoglutarate and L-aspartate react, which is catalyzed by AST. The enzyme malate dehydrogenase (MDH) oxidizes NADH to NAD and changes oxaloacetate to malate. The drop in NADH absorbance at 340 nm is directly correlated with the level of AST in the blood. It is a reaction with a kinetic rate (2).


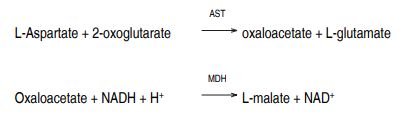


- 1. Alkaline phosphatase (ALP)

Test Kit name: ALP2

Purpose

There are four structural genotypes of serum alkaline phosphatase: intestine, placental, liver-bone-kidney, and the germ cell variety. The kidneys, spleen, placenta, prostate, small intestine, hepatocytes, leukocytes, and osteoblasts all contain the enzyme. Clinically, the liver-bone-kidney type is the most significant. In all types of cholestasis, but especially in obstructive jaundice, there is an increase in serum alkaline phosphatase. In addition, it is increased in malignant tumors, fractures, and bone illnesses such as osteomalacia, rickets, hyperparathyroidism, and Paget's disease. Following accelerated bone growth, children and juveniles may also show large increases in serum alkaline phosphatase levels due to enhanced osteoblast activity (3).

Principles

ALP uses a straightforward reaction in which alkaline phosphatase reacts with a substrate (p-nitrophenol phosphate, or PNPP) in the presence of activators for magnesium and zinc to produce a colorful product 34 (p-nitrophenol), which is visible at 450 nm. A direct proportion is found between the quantity of alkaline phosphatase present in the samples and the rate at which p-nitrophenol is formed (4).


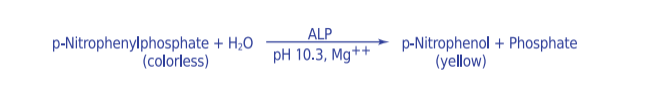


- 1. Total protein

Test kit name: TP2

Purpose

Hypoproteinemia, or low protein levels, can result from conditions like blood loss, sprue, nephrotic syndrome, severe burns, salt retention syndrome, or Kwashiorkor. The albumin/globulin (A/G) ratio helps track the balance between these protein fractions, and significant changes can appear in liver cirrhosis, glomerulonephritis, nephrotic syndrome, hepatitis, lupus, and other acute or chronic inflammations. Measuring total protein is valuable for diagnosing and managing diseases affecting the liver, kidneys, bone marrow, or overall nutrition and metabolism.

Principles

The total protein method utilizes the biuret reaction, with measurement of the final product at 546 nm. Divalent copper reacts in alkaline solution with protein peptide bonds to form the characteristic purple-colored biuret complex. Sodium potassium tartrate prevents the precipitation of copper hydroxide, and potassium iodide prevents auto-reduction of copper. The color intensity is directly proportional to the protein and measured photometrically at 546nm (5).


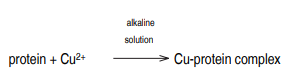


- 1. **. Albumin**

**Test kit name: ALB2**

**Purpose**

Numerous illnesses can result in hypoalbuminemia, which can be caused by several factors, including impaired synthesis from liver disease or decreased protein absorption; increased catabolism from inflammation or tissue damage (such as severe burns); malabsorption of amino acids (Crohn's disease); proteinuria from nephrotic syndrome; and protein loss through the stool (neoplastic disease). The highest plasma albumin content in severe cases of hypoalbuminemia is 2.5 g/dL. Water permeates into tissue through blood capillaries as a result of the plasma's low osmotic pressure (edema). The measurement of albumin is a great indicator of liver function and permits the tracking of a patient's dietary supplements (6).

Principles

Albumin shows a sufficiently cationic nature at pH 4.1 to form a blue-green complex with the anionic dye bromcresol green (BCG). The blue-green color intensity is measured photometrically at 570 nm and is directly proportional to the amount of albumin present in the sample (7).


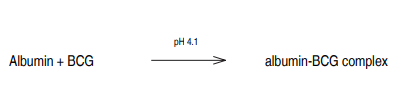


- 1. **Total bilirubin (TBL)**

Test Kit name: BILT3

Purposes

Unconjugated (indirect) bilirubin levels rise in the bloodstream when diseases or disorders that create bilirubin more quickly than the liver can metabolize it through hemolytic mechanisms do so. Due to comparable increases in circulating unconjugated bilirubin, liver immaturity, and several other illnesses in which the bilirubin conjugation pathway is compromised. Conjugated (direct) and unconjugated (indirect) bilirubin levels in the blood rise as a result of bile duct blockage or hepatocellular injury. (8).

Principles

It worked based on the diazo reaction. Total bilirubin, in the presence of a suitable solubilizing agent, is coupled with 3, 5-dichlorophenyl diazonium in a strongly acidic medium. The color intensity of the red azo dye formed is directly proportional to the total bilirubin and can be determined photometrically at 546 nm (9).


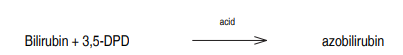


- 1. **Direct Bilirubin (DBL)**

Test kit name: BILD2

Principles

Conjugated bilirubin and δ‑bilirubin (direct bilirubin) react directly with 3,5 Dichlorophenyl diazonium salt in an acid buffer to form the red‑colored azobilirubin. The color intensity of the red azo dye formed is directly proportional to the direct (conjugated) bilirubin concentration and can be determined photometrically at 546 nm (10).


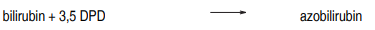


1. **Butrylcholinesterase (BChE)**

**Test Kit name: CHE2**

**Purpose**

The liver, pancreas, heart, serum, and white matter of the brain all contain cholinesterase, sometimes known as pseudo-cholinesterase or cholinesterase II. Cholinesterase's biological function is unknown. An indication of potential insecticide toxicity is serum cholinesterase. It is quantified as a liver function index. Intoxication with organophosphorus chemicals, cirrhosis, myocardial infarction, hepatitis, acute infections, and abnormal enzyme phenotypes are all associated with decreased cholinesterase levels (11).

Principles

This assay is based on the method published by Schmidt et al (11). Cholinesterase catalyzes the hydrolysis of butyrylthiocholine to thiocholine and butyrate. Thiocholine instantaneously reduces the yellow hexacyanoferrate (III) to the almost colorless hexacyanoferrate (II). This decrease in color can be measured photometrically. The decrease in absorbance due to the conversion of hexacyanoferrate (III)into hexacyanoferrate (II), and proportional to BChE activity in the specimen, is measured at 405 nm.


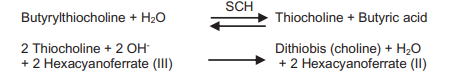


**Interpretation of results**

The result was interpreted by using the normal reference range of test kits as follows. Therefore, after the test had been analyzed, the result for each analyte was interpreted based on the Adult reference range.

Table1: Reference ranges of liver function, renal function, and BChE

| S. No | Tests | Sex | Reference ranges |
| --- | --- | --- | --- |
| 1 | ALT | Adult Male | <41 U/L |
|  |  | Adult Female | <33 U/L |
| 2 | AST | Adult Male | <40 U/L |
|  |  | Adult Female | <31 U/L |
| 3 | ALP | Adult Male | 40-129 U/L |
|  |  | Adult Female | 30-104 U/L |
| 4 | Total protein | Adult | 6.6 -8.7 g/dl(66-87 g/l) |
| 5 | Albumin | Adult | 3.5-5.2 g/dl (35-52 g/l) |
| 6 | Globulin | Adult | 2- 3.5 g/dl (20-35 g/l) |
| 7 | TBL | Adult Male | <1.4 mg/dl |
|  |  | Adult Female | 0.9 mg/dl |
| 8 | DBL | Adult | <0.30 mg/dl |
| 9 | BChE | Adult Male | 5320-12920 U/L |
|  |  | Adult Female | 4260-11250 U/L |
| 10 | A/G ratio | Adult | 1.0 - 2.0 |

1. Moss D, Henderson A, Kachmar J. Enzymes in: Tietz NW, ed. Fundamentals of clinical chemistry. Philadelphia: WB Saunders; 1987.

2. Bergmeyer H, Horder M, Rej R. Approved recommendation (1985) on IFCC methods for the measurement of catalytic concentration of enzymes. Part 2. IFCC method for aspartate aminotransferase (L-aspartate: 2-oxoglutarate aminotransferase, EC 2.6. 1.1). Journal of Clinical Chemistry and Clinical Biochemistry. 1986;24(7):497-508.

3. Bessey OA, Lowry OH, Brock MJ. A method for the rapid determination of alkaline phosphatase with five cubic millimeters of serum. Journal of biological chemistry. 1946;164(1):321-9.

4. Schumann G, Klauke R, Canalias F, Bossert-Reuther S, FH Franck P, Gella F-J, et al. IFCC primary reference procedures for the measurement of catalytic activity concentrations of enzymes at 37 C. Part 9: Reference procedure for the measurement of catalytic concentration of alkaline phosphatase: International Federation of Clinical Chemistry and Laboratory Medicine (IFCC) Scientific Division, Committee on Reference Systems of Enzymes (C-RSE) 1. Clinical chemistry and laboratory medicine. 2011;49(9):1439-46.

5. Weichselbaum CT. An accurate and rapid method for the determination of proteins in small amounts of blood serum and plasma. American journal of clinical pathology. 1946;16(3_ts):40-9.

6. GRANT GH. Amino acids and proteins. Fundamentals of clinical chemistry. 1987.

7. Doumas BT, Watson WA, Biggs HG. Albumin standards and the measurement of serum albumin with bromcresol green. Clinica chimica acta. 1971;31(1):87-96.

8. Balistreri W. Liver function. Fundamentals of clinical chemistry. 1987:729-61.

9. AW W. Modification of the Malloy-Evelyn method for a simple, reliable determination of total bilirubin in serum. Scand J Clin Lab Invest. 1972;29:11-2.

10. Malloy HT, Evelyn KA. The determination of bilirubin with the photoelectric colorimeter. Journal of biological Chemistry. 1937;119(2):481-90.

11. Schmidt E. Proposal of standard methods for the determination of enzyme catalytic concentrations in serum and plasma at 37℃, II. Cholinesterase. Eur J Clin Chem Clin Biochem. 1992;30:163-70.
